# Analysis of sodium self-inhibition of the epithelial sodium channel (ENaC) using classical and medium-throughput automated two-electrode voltage-clamp recordings

**DOI:** 10.1007/s00424-026-03212-w

**Published:** 2026-09-28

**Authors:** Karen Lizet Luján López, Nadja Furtwängler, Carol Merlin Frank, Mike Althaus, Oliver Rauh

**Affiliations:** 1 Institute for Functional Gene Analytics, Bonn-Rhein-Sieg University of Applied Sciences, Von-Liebig-Str. 20, Rheinbach, 53359 Germany; 2 Institute for Animal Physiology, Justus-Liebig-University, Giessen, Germany

**Keywords:** Epithelial Sodium Channel, ENaC, Sodium, Sodium self-inhibition, Two-electrode voltage-clamp, Automated electrophysiology

## Abstract

The epithelial sodium channel (ENaC) is a key regulator of electrolyte homeostasis and blood pressure. A characteristic ENaC feature is sodium self-inhibition (SSI), a rapid autoregulatory mechanism in which extracellular Na^+^ ions allosterically bind ENaC and facilitate transition into a low open probability state. Although SSI is an important functional parameter for assessing ENaC activity and gating, its quantification has traditionally relied on manual two-electrode voltage-clamp (TEVC) recordings with limited scalability and throughput. Here, we improved and evaluated experimental strategies and analytical methods for robust quantification of ENaC SSI in manual and automated TEVC systems: (1) The determination of SSI from extrapolation of transient current recordings in response to a jump in the extracellular Na^+^ concentration; (2) the estimation of SSI by comparison of steady-state currents before and after chemical modification of a β_S520C_-ENaC mutant with MTSET; and (3) quantification of SSI from the analysis of steady-state current-voltage relationships recorded at different extracellular Na^+^ concentrations. The latter procedure is the most reliable for SSI quantification in perfusion rate-limited automated TEVC systems. These methods can expand the capabilities of automated electrophysiology and support medium- to high-throughput investigation of autoregulatory mechanisms in ENaC variants and the discovery of novel ENaC modulators.

**Supplementary Information:**

The online version contains supplementary material available at https://doi.org/10.1007/s00424-026-03212-w.

## Introduction

Ion channels play a crucial role in many physiological processes, and their dysfunction is associated with human diseases (channelopathies). Ion channels are therefore highly important targets for molecular drug research and represent the second largest target for existing pharmaceuticals, after G protein-coupled receptors [2, 37]. Conventional electrophysiological techniques, such as patch-clamp and microelectrode recordings, have been employed for decades to advance the understanding of ion channel biophysics, physiology, pathophysiology, and pharmacology. However, the time-consuming nature of these techniques has limited large-scale drug screening and development efforts. The development of medium- to high-throughput electrophysiological techniques, such as automated planar patch-clamp recordings [16, 21, 52] and automated two-electrode voltage-clamp (TEVC) recordings using *Xenopus laevis* oocytes [35, 47, 55], in combination with modern AI-driven approaches in molecular drug discovery and design [49, 64], have paved the way for the development of novel ion channel targeting compounds. Nevertheless, specific ion channel characteristics, such as fast gating kinetics, desensitisation or sensitivity to experimental conditions, remain challenges for functional medium- to high-throughput assays.

An ion channel with high clinical and pharmacological relevance is the epithelial sodium channel (ENaC). ENaC is a voltage-insensitive, constitutively active, sodium-selective heterotrimeric ion channel which is part of the degenerin/ENaC protein family [39, 53]. ENaC can exist in different assemblies of its four subunits (α, β, γ and δ) [40], with αβγ-ENaC [10] and δβγ-ENaC [60] generating robust sodium currents which are blocked by the diuretic amiloride. Canonical (αβγ-)ENaC is present in the apical membrane of various epithelia including those of the aldosterone-sensitive distal nephrons in the kidneys, the colon, airways and alveoli, middle ear, sweat glands, as well as the reproductive tract [53]. In these epithelia, ENaC represents the rate-limiting step for transepithelial sodium, and consequently, water re-absorption and therefore plays a key role in electrolyte and fluid homeostasis [53]. In the kidneys, ENaC also controls renal potassium excretion by providing a lumen-negative potential across the apical principal cell membrane, thereby driving potassium efflux via the renal outer medullary K^+^ channel (ROMK) [46]. Furthermore, canonical and non-canonical ENaC assemblies have been implicated in sensory functions in taste buds, vascular endothelial cells, immune cells and the brain [28]. These diverse physiological functions require precise control of ENaC activity which is achieved by glucocorticoid- and mineralocorticoid-controlled expression of subunit-coding genes, a network of kinases regulating trafficking to and removal from the membrane, as well as control of open probability (P_O_) by intra- and extracellular stimuli (for excellent reviews see [28, 46, 53]. Deregulated ENaC activity results in severe human diseases. For example, gain-of-function mutations in ENaC subunit-coding genes cause Liddle syndrome [1, 54] a hereditary form of hypertension and concomitant hypokalaemia, while loss-of-function mutations cause pseudo-hypoaldosteronism type 1 [13, 18, 53], characterised by renal salt-wasting, hypotension and hyperkalaemia. Furthermore, aberrant ENaC regulation is implicated in the pathophysiology of Conn’s syndrome (primary aldosteronism) [32], diseases associated with secondary hyperaldosteronism such as congestive heart failure and cirrhosis [7], nephrotic syndrome [22], salt-sensitive hypertension, cystic fibrosis [50], and acute respiratory distress syndrome [61].

ENaC has unique properties that determine its P_O_ and make its functional analysis in medium- to high-throughput automated electrophysiology systems challenging. For example, the channel’s α- and γ-subunits contain inhibitory peptides embedded within the extracellular ‘Gating Release of Inhibition by Proteolysis’ (GRIP) domains [12, 39, 40, 45] which lock ENaC in a low P_O_ state [5]. The α-ENaC inhibitory peptide is flanked by furin cleavage sites and is therefore released by furin-mediated cleavage within the *trans*-Golgi network. By contrast, there is only a single furin cleavage site proximal to the γ-ENaC inhibitory peptide which is therefore not completely released during this step. Furin-processed channels exhibit an intermediate P_O_ and are delivered to the plasma membrane [24]. Distal to the γ-ENaC inhibitory peptide, several motifs are present that are recognised by various extracellular proteases, including membrane-anchored proteases such as prostasin or soluble proteases including chymotrypsin or trypsin [3]. Cleavage of γ-ENaC causes full release of its inhibitory peptide, resulting in a marked increase in P_O_ [11]. Notably, many of the proteases capable of activating ENaC via this mechanism are commonly used in cell culture protocols. This necessitates careful evaluation of the proteolytic state of ENaC populations present at the cell membrane during electrophysiological recordings and suitable experimental protocols for automated planar patch-clamp recordings have recently been established [58].

Another characteristic ENaC feature is the sensitivity of P_O_ to the extracellular Na^+^ concentration ([Na^+^]_ex_). This was first observed in transepithelial ion transport recordings using frog skin preparations in Ussing chambers, where a rapid rise in apical [Na^+^]_ex_ caused a short increase in transepithelial sodium absorption which subsequently declined to a lower steady-state level [17]. Later studies on heterologously expressed ENaCs in *X. laevis* oocytes [27] as well as cryo-electron microscopy derived ENaC structures [40] revealed that sodium ions bind to an acidic region formed by the extracellular β6-β7 loop of the α-subunit. Na^+^ binding to this region leads to a decrease in P_O_ by promoting the transition into the closed state, a mechanism referred to as sodium self-inhibition (SSI). This allosteric control of ENaC P_O_ by Na^+^ was suggested to adjust ENaC activity to fluctuating [Na^+^]_ex_ which, for example, can contribute to fine-tuning of sodium re-absorption and potassium excretion in the renal distal nephron [28]. SSI is not only an important physiological mechanism controlling ENaC activity in response to [Na^+^]_ex_, it also provides information on biophysical channel properties as the time course of the current decay in response to high [Na^+^]_ex_ is indicative of the transition kinetics between low and high P_O_ states and can provide relevant insights into ENaC gating.

Unfortunately, there are currently no established protocols that allow precise quantification of ENaC SSI in medium- to high-throughput electrophysiology systems [33]. In general, SSI can be observed in electrophysiological recordings, but a careful experimental design is essential since Na^+^ ions are both the signal- (i.e. current) generating factor and the factor facilitating allosteric ENaC inhibition. The current gold-standard for SSI quantification are manual TEVC recordings on ENaC-expressing *Xenopus* oocytes, where the [Na^+^]_ex_ is rapidly switched from a low [Na^+^]_ex_ at which ENaC P_O_ is high, to a high [Na]_ex_ which then facilitates the allosteric Na^+^-mediated transition to the lower P_O_ state. The [Na^+^]_ex_-jump therefore causes a transient current response representing SSI, but this transient current is only visible when the buffer exchange rate of the employed TEVC system is faster than the allosteric ENaC inhibition by Na^+^. This is particularly challenging to detect in microplate-based automated TEVC systems. We therefore compared a commercially available automated TEVC system to a manual fast-perfusion TEVC system and systematically evaluated the suitability of both to accurately detect ENaC SSI. We developed experimental strategies and analytical methods that can be applied to overcome limitations of automated TEVC recordings and enable medium- to high-throughput quantification of this key ENaC autoregulatory feature.

## Materials and methods

### Plasmids and site-directed mutagenesis

Coding sequences of human ENaC subunits (α-, β-, γ-ENaC) were present in the pTNT vector (Promega, Walldorf, Germany). The National Center for Biotechnology Information (NCBI) reference numbers of the coding sequences are NM_001038 (α-ENaC), NM_000336 (β-ENaC) and NM_001039 (γ-ENaC). The α-ENaC subunit contained a frequent polymorphism at position 663 (p.Thr663Ala; dbSNP reference rs2228576).

To generate a mutation in the conserved degenerin site of the human β-ENaC subunit [19], the serine residue at position 520 was substituted by cysteine (p.Ser520Cys) using the Q5 Site-directed Mutagenesis Kit (New England Biolabs, Frankfurt am Main, Germany) according to the manufacturer’s instructions. Mutagenesis primer sequences were (forward) 5’-CTGGCTGCTCtgcAATCTGGGTG-3’ and (reverse) 5’- ACGATGTTATTGGCTGC-3’. The mutated plasmids were introduced into rubidium chloride competent DH5α *E. coli* via heat-shock transformation at 42 °C. Plasmids were isolated using the Monarch Plasmid Miniprep Kit (New England Biolabs) according to the manufacturer’s instructions and eluted in nuclease-free water. The S520C substitution was confirmed via Sanger sequencing (Eurofins Genomics, Ebersberg, Germany). Plasmids were subsequently purified with the Monarch PCR and DNA Cleanup Kit (New England Biolabs) according to manufacturer’s instructions, eluted in nuclease-free water and stored at −20 °C until further use.

### cRNA synthesis and heterologous expression in *Xenopus laevis* oocytes

Cleaned plasmids were used as template DNA for cRNA synthesis using the HiScribe T7 ARCA mRNA Kit (New England Biolabs) according to the manufacturer’s instructions and cRNA was eluted in nuclease-free water. cRNA coding for α-, β- (or β_S520C_-), and γ-ENaC were combined at a concentration of 5 ng/µl per subunit in nuclease-free water and stored at −80 °C.

For heterologous expression, *X. laevis* oocytes (stage V/VI) were purchased from Ecocyte Bioscience (Dortmund, Germany). Upon arrival, oocytes were stored at 4 °C in Modified Barth´s Solution (MBS; containing in mM: 88 NaCl, 1 KCl, 2.4 NaHCO_3_, 0.82 MgSO_4_, 0.33 Ca(NO_3_)_2_, 0.41 CaCl_2_ and 10 HEPES; pH = 7.5 adjusted with NaOH) supplemented with 20 µg/ml of gentamycin. For microinjection, oocytes were placed in a low-sodium Oocyte Ringer’s Solution (low-sodium ORS; containing in mM: 10 NaCl, 1 KCl, 2 CaCl_2_, 2.5 Sodium pyruvate, 80 NMDG and 5 HEPES; pH = 7.4 adjusted with HCl) supplemented with 20 µg/ml of gentamycin. Oocytes were injected with 18.4 nl of the previously combined αβγ-ENaC cRNA using the Nanoject II or Nanoject III Microinjectors (Drummond Scientific Company, Broomall, PA, USA). For [Na^+^]_ex_-jump experiments performed using the automated TEVC system, a cRNA concentration of 5 ng/µl was used. For all other manual and automated TEVC recordings, a lower concentration of 0.5 ng/µl was used to reduce ENaC-mediated Na^+^ current. This was necessary due to the characteristics of the amplifier used in manual TEVC recordings and in order to limit the accumulation of intracellular Na^+^ and, consequently, ENaC current run-down during longer recordings with the automated TEVC system (e.g. to determine steady-state I/V relationships). After injection, oocytes were incubated in low-sodium ORS at 16 °C for 24 h prior to electrophysiological recordings.

### Manual two-electrode voltage-clamp recordings

**Fig. 1:**
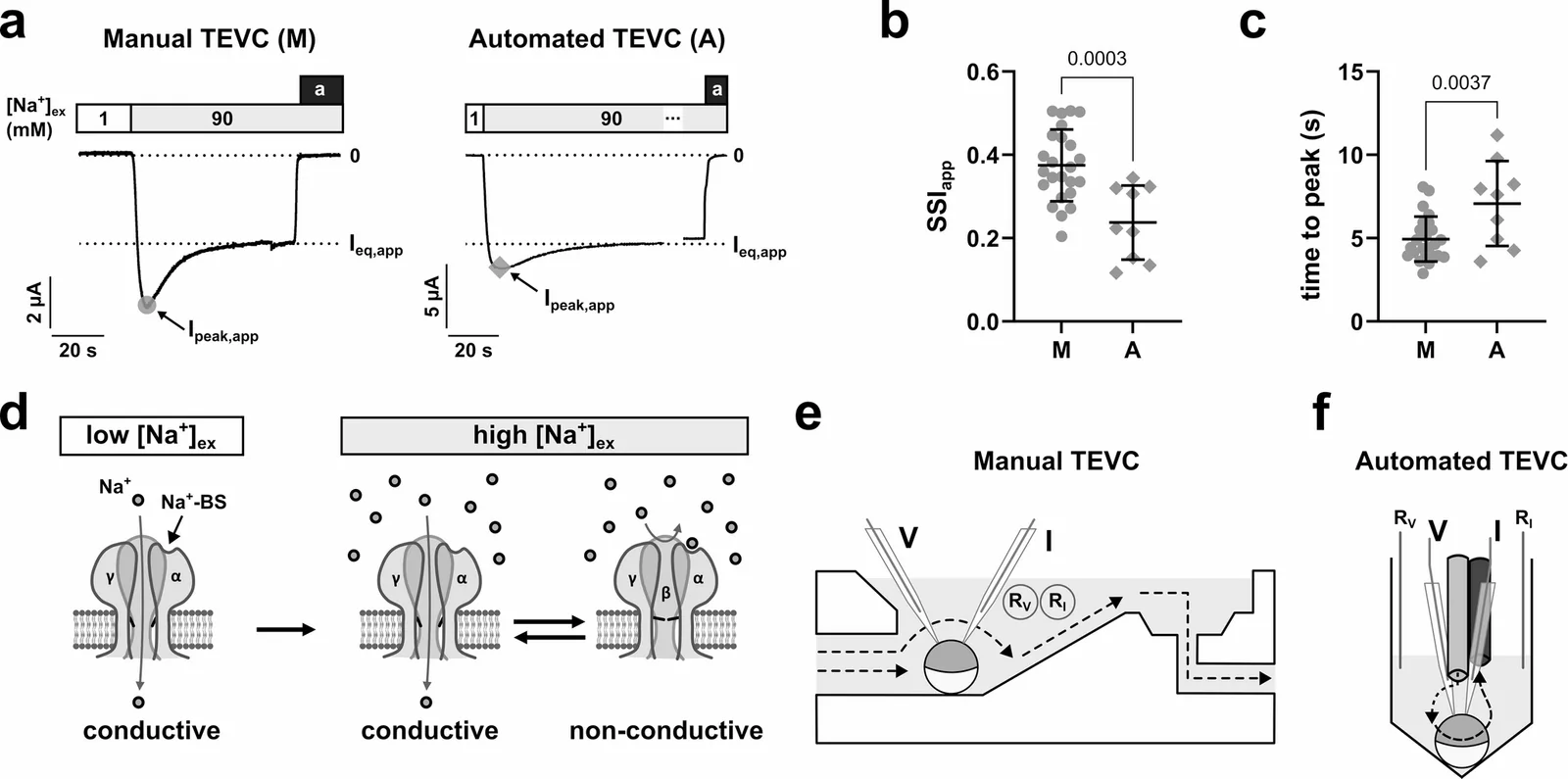
Comparison of current responses and SSI from [Na^+^]_ex_-jump experiments performed using manual and automated TEVC. (**a**) Representative transmembrane current (I_M_) traces from manual (M, left) and automated (A, right) TEVC recordings at V_M_ = −60 mV of oocytes expressing αβγ-ENaC. [Na^+^]_ex_ was changed from 1 mM to 90 mM; 100 µM amiloride (a; black box) was applied at the end of each recording. I_peak,app_ indicates the apparent peak current after the [Na^+^]_ex_-jump and I_eq,app_ the apparent equilibrium current measured 60 s after the [Na^+^]_ex_-jump. (**b**) Comparison of apparent sodium self-inhibition (SSI_app_) from manual (M; *n* = 24, *N* = 6), and automated (A; *n* = 9, *N* = 3) TEVC recordings as in (a). SSI_app_ was calculated from I_peak,app_ and I_eq,app_ with Eq. 23. Statistical analysis was performed using Student’s unpaired t-test. (**c**) Comparison of time-to-peak values from manual (M) and automated (A) TEVC recordings as in (a). Individual time-to-peak values were measured as the time between the onset of current rise (rising phase) following the [Na^+^]_ex_-jump and the time of I_peak, app_. Data points in (b) and (c) represent data from n individual experiments; lines and error bars represent arithmetic mean ± SD. Statistical analysis was performed using Student’s unpaired t-test with Welch’s correction. (**d**) Simplified schematic depiction of αβγ-ENaC’s conductive and non-conductive states depending on the extracellular sodium concentration [Na^+^]_ex_. With low [Na^+^]_ex_, the α-subunit’s Na^+^ binding site (Na^+^-BS) remains empty most of the time, causing the channel to be primarily in the Na^+^-conductive state. With high [Na^+^]_ex_, the Na^+^-BS is primarily occupied, which promotes transition into the non-conductive state. The steady-state open probability is determined by the transition frequencies between the conductive and non-conductive states. (**e**,** f**) Schematic representations of perfusion chambers in the employed manual (**e**) and automated (**f**) TEVC systems. V = voltage electrode; I = current electrode; R_v_, R_I_ = reference electrodes for V and I. Dashed arrows provide a simplified illustration of fluid movement during perfusion

For manual two-electrode voltage-clamp (TEVC) recordings, ENaC-expressing oocytes were placed in a custom-made recording chamber (Fig. 1e) filled with 1 mM Na^+^ solution (for composition see below). The chamber allowed fast gravity-driven perfusion (20 ml/min) and perfusion speed was measured prior to the beginning of each day of recording. Chlorided silver wires served as recording electrodes and were inserted into micropipettes pulled from borosilicate glass capillaries (1B120-4; World Precision Instruments; Sarasota, FL, USA) using a DMZ Universal Puller (Zeitz, München, Germany) and backfilled with 3 M KCl. Resistance of the electrodes ranged between 0.1 and 1 MΩ. To minimise voltage offset errors, 3 M KCl 3% Agar bridges were used to electrically connect the bath electrodes to the bath solution. Oocyte whole-cell transmembrane currents (I_M_) were recorded using an OC-725B voltage-clamp amplifier (Warner Instruments Corp., Hamden, CT, USA), at a holding potential of −60 mV during the whole experiment. Currents were digitised by an AD/DA interface (Axon Digidata 1550B plus HumSilencer, Molecular Devices, Wokingham, United Kingdom), filtered at 100 Hz using a 4-pole low pass Bessel filter (Warner Instruments Corp.) and recorded with pClamp v.11.4.3 (Molecular Devices) at a 1 kHz sampling rate.

### Automated two-electrode voltage-clamp recordings

For automated TEVC recordings, ENaC-expressing oocytes were washed with 1 mM Na^+^ solution before being placed in a V-shaped 96 well microtiter plate (ThermoFisher Scientific, Waltham, MA, USA) filled with 200 µL of 1 mM Na^+^ solution. Oocyte whole-cell transmembrane currents (I_M_) were recorded using the Roboocyte2 device alongside its corresponding measuring head (Fig. 1f) containing the recording and bath electrodes (Multi Channel Systems, Reutlingen, Germany). Ag/AgCl wires served as recording electrodes and were inserted into micropipettes pulled from borosilicate glass capillaries (TW150-4; World Precision Instruments) using a DMZ Universal Puller (Zeitz) and backfilled with 3 M KCl. Resistance of the recording electrodes was between 0.05 and 2 MΩ. After impalement, oocytes were clamped to −60 mV. Currents recorded in [Na^+^]_ex_-jump experiments were digitised with a sampling frequency of 20 Hz. Current–voltage (I/V) relationships were obtained using a voltage-step protocol in which the membrane potential was stepped from a holding potential of − 60 mV to test potentials ranging from − 120 to + 50 mV in 10 mV increments. Each voltage step was applied for 200 ms and recorded currents were digitised with a sampling frequency of 100 Hz. Current and voltage signals were recorded using the Roboocyte2+ software (Multi Channel Systems). Perfusion of recording solutions was performed using an integrated pinch valve Roboflow-System (Multi Channel Systems). The valve pump speed was optimised to a perfusion rate of ~ 5 ml/min which yielded the most stable recordings. Furthermore, in order to enhance the rate of solute exchange near the oocyte, the perfusion inflow tubing of the measuring probe was slightly altered to produce a wider opening that directed the perfusion solution flow directly towards the oocyte.

### Recording solutions

Protocols for both the manual and automated TEVC recordings used the same external bath solutions: a 1 mM Na^+^ solution (in mM: 1 NaCl, 1 KCl, 2 CaCl_2_, 89 NMDG and 5 HEPES; pH = 7.4 adjusted with HCl); a 10 mM Na^+^ solution (in mM: 10 NaCl, 1 KCl, 2 CaCl_2_, 80 NMDG and 5 HEPES; pH = 7.4 adjusted with HCl); and a 90 mM Na^+^ solution (in mM: 90 NaCl, 1 KCl, 2 CaCl_2_ and 5 HEPES; pH = 7.4 adjusted with NaOH). The ENaC blocker amiloride was prepared as a 10 mM stock solution in distilled water and used at a final concentration of 100 µM in the corresponding external solution. Solutions containing MTSET were prepared immediately before use by dissolving MTSET at a final concentration of 1 mM in 1 mM Na^+^ or 90 mM Na^+^ solutions. Chymotrypsin was prepared immediately before use as a 2 mg/ml stock solution in distilled water and used at a final concentration of 2 µg/ml in 1 mM Na^+^ solution. All recording solutions were kept at room temperature prior to recording.

### Chemicals

CaCl₂, Ca(NO₃)₂, HCl, MgSO₄, NaCl, NaHCO₃, NaOH and chymotrypsin were from Carl Roth (Karlsruhe, Germany). 2-[4-(2-hydroxyethyl)piperazin-1-yl]ethanesulfonic acid (HEPES) and KCl were from Fisher BioReagents (Thermo Fisher Scientific, Waltham, MA, USA). Gentamycin (10 mg/ml) and sodium pyruvate were from Gibco (Thermo Fisher Scientific, Waltham, MA, USA). Amiloride hydrochloride and N-methyl-D-glucamine (NMDG) were from Sigma-Aldrich (Merck KGaA, Darmstadt, Germany). 2-(trimethylammonium)ethyl methanethiosulfonate, bromide (MTSET) was from Biotium (Fremont, CA, USA).

### Simulation of ENaC currents

Na^+^-dependent gating of ENaC is modelled using a two-state Markov-model:

$$\:A\begin{array}{c}{k}_{AI}\\\:\:\rightleftharpoons\:\:\\\:{k}_{IA}\end{array}I$$

In this model, ENaC can exist in an active (Na^+^-conductive) state *A*, and a Na^+^-inhibited (non-conductive) state *I*. The transitions between these two states are determined by the inhibition rate constant *k*_*AI*_ and the activation rate constant *k*_*IA*_ with *k*_*AI*_ depending linearly on [Na^+^]_ex_ (Eq. 24). It must be noted that the linear relationship between k_AI_ and [Na^+^]_ex_ is a simplification; a strictly linear relationship is only to be expected between k_AI_ and the Na^+^ activity.

It is also important to mention that state A does not necessarily have to be identical to the open state O. State A should rather be considered a pseudo-state which may include one or more closed states in addition to the open state. Although the model predicts an active-state probability of P_A_ = 1 at [Na^+^]_ex_ = 0, the open probability may nevertheless differ significantly from 1.0. In fact, Sheng and colleagues demonstrated that a furin cleavage site mutant of human αβγ-ENaC has an apparent SSI value of ~ 0.8 (determined from transient current recordings in response to an [Na^+^]_ex_-jump from 1 to 110 mM) which is caused by a reduction in the single-channel open probability from 0.38 ± 0.06 at [Na^+^]_ex_ = 1 mM to 0.07 ± 0.02 at [Na^+^]_ex_ = 110 mM [56]. This illustrates that the open probability within the active state (i.e. the conditional open probability P(O|A)) may depend on other intra- or extracellular regulatory factors, e.g. intracellular Na^+^ or mechanical forces [53], and that SSI always reflects relative changes in P_O_.

The model predicts that a sudden change in the extracellular Na^+^ concentration ([Na^+^]_ex_) leads to a time-dependent change in the probability to find a channel in the active state:

1 $$\:P_A\left(t\right)={P_{A,eq}+\left(P_A\left(0\right)-P_{A,eq}\right)\cdot\:e}^{-\frac t{{\tau}_{true}}}$$

with P_A_(0) and P_A, eq_ being the probability that the channel is in the active state at t = 0 and in equilibrium (t = ∞), respectively. For details see Appendix S1. The exponential time constant τ_true_ and the equilibrium probability are given by:

2 $$\:{\tau\:}_{true}=\frac1{{k'}_{AI}\cdot\:{\left[{Na}^+\right]}_{ex}+k_{IA}}$$

and

3 $$\:P_{A,eq}=\frac{k_{IA}}{{k'}_{AI}\cdot\:{\left[{Na}^+\right]}_{ex}+k_{IA}}$$

**Fig. 2:**
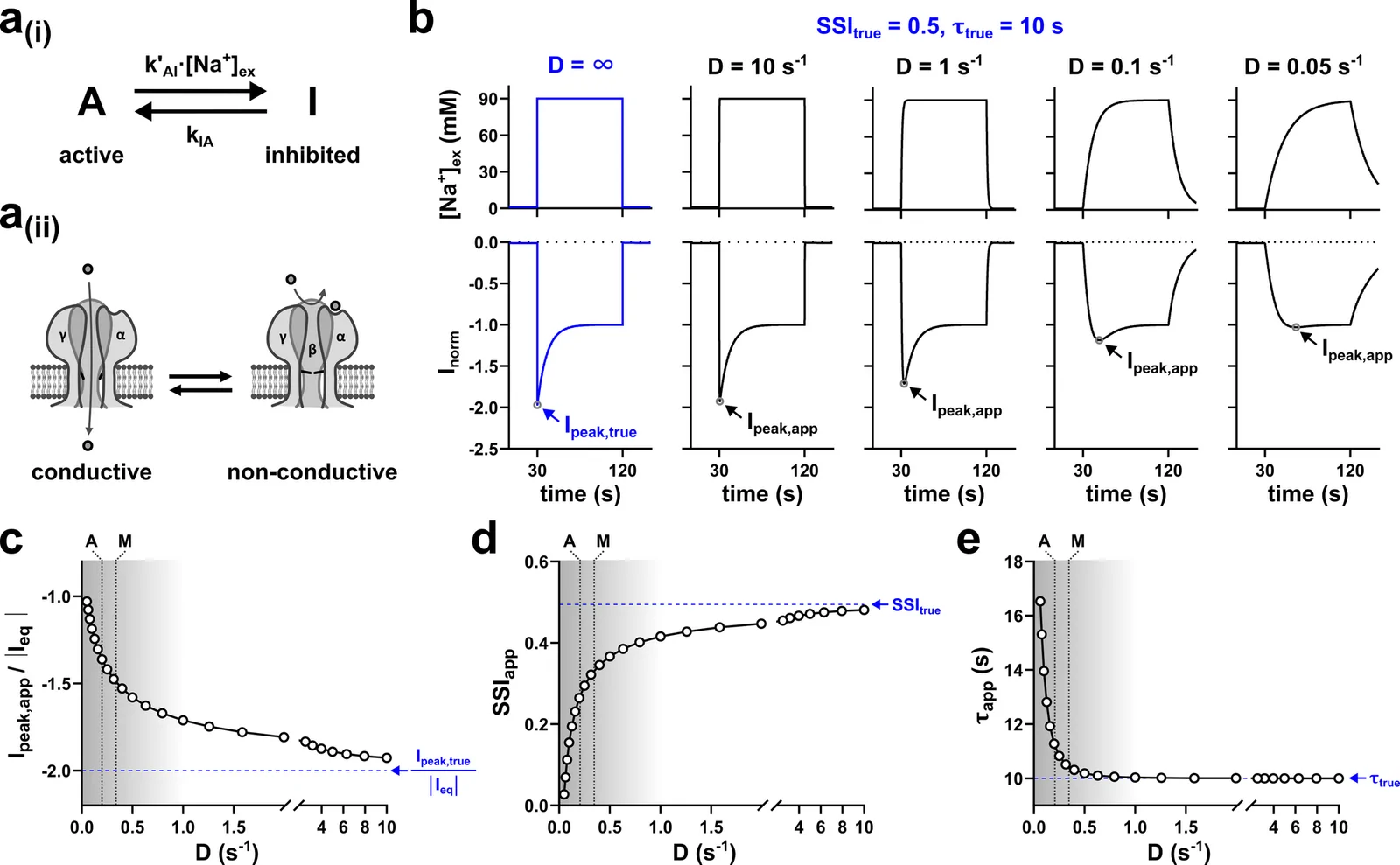
Simulation of [Na^+^]_ex_-jump experiments reveals that SSI_app_ is highly sensitive to the perfusion rate.(**a**) Two-state Markov-model (**a**_(i)_) used to simulate the [Na^+^]_ex_-dependency of ENaC activity. ENaC can exist in an active state (A) and in a Na^+^-inhibited state (I). The transition from A to I is defined by the [Na^+^]_ex_-dependent rate constant k_AI_ = k’_AI_∙[Na^+^]_ex_. The transition from I to A is defined by the [Na^+^]_ex_-independent rate constant k_IA_. In the active state A, the channel pore is conductive and allows Na^+^ permeation. In the inhibited state I, the pore is non-conductive and does not allow Na^+^ permeation. An illustration of this model is shown in (**a**_(ii)_). (**b**) Numerically simulated [Na^+^]_ex_-profiles (top row) in a TEVC recording chamber during a [Na^+^]_ex_-jump experiment and corresponding normalised ENaC-mediated current responses at V_M_ = −60 mV (bottom row) for different perfusion rates D. The [Na^+^]_ex_-profiles were calculated using a continuous stirred tank reactor model. In all simulations, the Na^+^ concentration of the inflowing solution was increased after 30 s from 1 mM to 90 mM. 90 s after this [Na^+^]_ex_-jump, the concentration was reduced back to 1 mM. Current traces were calculated using the two-state model shown in (a) and the Goldman-Hodgkin-Katz (GHK) flux model (Eq. 6) for SSI_true_ = 0.5, τ_true_ = 10 s, and intracellular [Na^+^]_in_ = 5 mM. Peak currents after the [Na^+^]_ex_-jump are highlighted with open grey circles. Currents were normalised to the equilibrium current at [Na^+^]_ex_ = 90 mM. (**c**) Perfusion-rate dependence of the ratio of apparent peak current (I_peak,app_) to absolute value of the equilibrium current at [Na^+^]_ex_ = 90 mM (|I_eq_|) obtained from numerical simulations as in (b). (**d**) Perfusion-rate dependence of the apparent SSI (SSI_app_) obtained from numerical simulations as in (b). (**e**) Perfusion-rate dependence of the apparent time constant (τ_app_) of the exponentially decaying part of the falling phase. Black connecting lines between the data points (open circles) in (c–e) represent linear interpolations. Values for D = ∞ (true values) are indicated in (c–e) by dashed lines and black arrows. The areas highlighted in grey in (c-e) indicate the range of experimentally feasible perfusion rates. Vertical dotted lines in (c-e) represent the mean values of the perfusion rates determined experimentally for the automated (A; D = 0.19 ± 0.11 s^− 1^) and manual (M; D = 0.33 ± 0.06 s^− 1^) TEVC systems

Substituting [Na^+^]_ex_ = 0 into Eq. 3 shows that the model in Fig. 2a_**(i)**_ predicts that, in the absence of external Na^+^, ENaC is in the active state *A* with a probability of 1. Accordingly, we define the true sodium self-inhibition (SSI_true_) at any external Na^+^ concentration as:

4 $${SSI}_{true\:}\mathrm{=}\:1-{P}_{A,eq}$$

To calculate the macroscopic current *I*, it was assumed that a channel conducts Na^+^ only when it is in state *A*. With *i* being the unitary current passing through a single channel in the open state O, the macroscopic current through *N* channels is given by:

5 $$I=i\cdot N\cdot P_O=i\cdot N\cdot P_A\cdot P\left(O\left|A\right.\right)$$

with P(O|A) being the conditional open probability given that the channel is in the active state A. Hence, P(O|A) is identical to the open probability at [Na^+^]_ex_ = 0 mM and can therefore be considered the baseline open probability. It is assumed that P(O|A) is a constant. If it is not a constant, it should be possible to experimentally detect a deviation from the linear dependence of k_AI_ on [Na^+^]_ex_. However, Chraïbi and Horisberger were able to show that k_AI_ increases for human ENaC almost linearly with [Na^+^]_ex_ in the concentration range between [Na^+^]_ex_ = 0 and 100 mM, which supports our assumption [14]. To calculate the unitary current *i* for given values of the membrane potential *V*_*M*_, the external Na^+^ concentration [Na^+^]_ex_, and the internal Na^+^ concentrations [Na^+^]_in_, we assume that the ion flux through the open ENaC pore can be approximated by the Goldman-Hodgkin-Katz (GHK) flux equation:

6 $$\begin{array}{c}\:i\left(V_M,{\left[{Na}^+\right]}_{ex},{\left[{Na}^+\right]}_{in}\right)\\=p_{Na}\cdot\:\frac{F^2}{R\cdot\:T}\cdot\:V_M\cdot\:\left(\frac{{\left[{Na}^+\right]}_{ex}-{\left[{Na}^+\right]}_{in}\cdot\:e^{\frac F{R\cdot\:T}\cdot\:V_M}}{1-e^{\frac F{R\cdot\:T}\cdot\:V_M}}\right)\end{array}$$

with p_Na_ being the single-channel Na permeability, F the Faraday constant (96485.3321 C/mol), R the universal gas constant (8.314 J/(K∙mol)), and T the absolute temperature. Simulations were performed for T = 298 K. Since all simulated current traces are displayed exclusively in normalised form, the values for p_Na_ and P(O|A) do not need to be specified, as they only act as scaling factors on the absolute currents.

To study experimentally the dependence of the ion channel activity on the [Na^+^]_ex_, [Na^+^]_ex_ is changed by perfusion. If a perfusion chamber is used, [Na^+^]_ex_ does not change abruptly. Rather, due to mixing and dilution effects, there is a time-dependent change in [Na^+^]_ex_ from the initial concentration [Na^+^]_ex_(0) to the desired concentration [Na^+^]_ex, eq_. To describe this change, it is assumed that the volume in the perfusion chamber remains constant during perfusion (dV/dt = 0) and that the mixing of the solution in the chamber occurs without any time delay (ideal continuous stirred tank reactor, ICSTR) [36]. Based on these assumptions, the change in [Na^+^]_ex_ during perfusion can be described with the following ordinary differential equation:

7 $$\text{ }\frac{d{\left[{Na}^+\right]}_{ex}}{dt}=\left({\left[{Na}^+\right]}_{ex,eq}-{\left[{Na}^+\right]}_{ex}\left(t\right)\right)\cdot\:D$$

with *D* being the perfusion rate, which is defined as the number of reactor volumes passing through the recording chamber per unit of time. Integration of Eq. 7 in the time interval [0,∞] gives:

8 $$\begin{array}{c}{\left[{Na}^+\right]}_{ex}\left(t\right)={\left[{Na}^+\right]}_{ex,eq}\\-\left({\left[{Na}^+\right]}_{ex,eq}-{\left[{Na}^+\right]}_{ex}\left(0\right)\right)\cdot\:e^{-D\cdot\:t}\end{array}$$

This equation makes it possible to calculate how the Na^+^ concentration in the perfusion chamber changes over time when a solution with a higher or lower Na^+^ concentration is flushed in with the specific perfusion rate *D*. The [Na^+^]_ex_ profiles generated using Eq. 8 exhibit an abrupt, non-continuous, change in slope at the moment of transition from one perfused solution (e.g. [Na^+^]_ex_ = 1 mM) to another (e.g. [Na^+^]_ex_ = 90 mM). Such discontinuities are not observed experimentally, probably due to additional mixing effects (e.g. in the tubing) which are not taken into account in the ICSTR model. To account for these more complex mixing phenomena which act like a low-pass filter on the [Na^+^]_ex_ profile around the oocyte, a heuristic approach was used: the concentration profiles computed with Eq. 8 were smoothed using a moving average filter with a window size of 100 ms, i.e. a cut-off frequency of 10 Hz. This frequency was determined empirically by comparing simulated and experimentally measured current traces.

All simulations were performed with V_M_ = −60 mV and [Na^+^]_in_ = 5 mM. This value for [Na^+^]_in_ is based on the mean intracellular Na^+^ activity of a_Na,in_ = 4.5 ± 1.0 mM, which we calculated from the reversal potentials (E_rev_) of a total of *n* = 23 amiloride-sensitive I/V relationships recorded at [Na^+^]_ex_ = 1 mM at the beginning of the experiments shown in Figs. 6 and 7. This a_Na,in_ corresponds to a concentration of approximately 5 mM. [Na^+^]_ex_ was changed during a simulation as described in the results section. Simulations were performed for SSI_true_ = 0.2 to 0.9 and τ_true_ = 1 to 10 s at [Na^+^]_ex_ = 90 mM. For each SSI_true_/τ_true_ combination, the rate constants were calculated as follows:

9 $$\:{k\operatorname{'}}_{AI}=\frac1{90\:mM}\cdot\frac{{SSI}_{true}}{{\tau}_{true}}$$

10 $$\:k_{IA}=\frac1{{\tau}_{true}}\cdot\left(1-{SSI}_{true}\right)$$

### Extrapolation method

Since the two-state model predicts that P_A_ relaxes exponentially after the [Na^+^]_ex_-jump (Eq. 1), the current amplitude during the falling phase can be described using the following exponential function:

11 $$\:I\left(t\right)\:=\left(I_{peak,ext}-I_{eq,ext}\right)\cdot\:e^{-\frac t{{\tau}_{app}}}+I_{eq,ext}$$

with I_peak, ext_ being the extrapolated peak current at t = 0, I_eq,ext_ the extrapolated equilibrium current, and τ_app_ the apparent time constant of the exponential current decay. Equation 11 was fitted to the current between the inflection point of the falling phase and the time at which about 80% of the current decay had occurred.

To account for current run-down caused by feedback inhibition [48, 59], it was assumed that the number of channels decreases approximately linearly over time with the rate constant *k*_*rd*_:

12 $$\begin{array}{c}\:I\left(t\right)\:=\left(\left(I_{peak,ext}-I_{eq,ext}\right)\cdot\:e^{-\frac t{{\tau}_{app}}}+I_{eq,ext}\right)\\\cdot\:\left(1-k_{rd}\cdot\:t\right)\end{array}$$

Eq. 12 was fitted to the recorded current between the inflection point of the falling phase and to a point about 60 s after the [Na^+^]_ex_-jump.

Since the solution exchange in the recording chamber is not instantaneous, t = 0 in Eqs. 11 and 12 is set to the inflection point of the rising phase. The inflection point was determined by fitting the current of the rising phase between the onset of the current rise (*L*_*1*_) and the point at which the current has reached approximately 70% of the peak current (*L*_*2*_) with a modified Hill equation:

13 $$\:I\left(t\right)\:\mathrm{=}\text{}\frac{\left({I}_{top}-{I}_{bottom}\right)}{\left(1+{\left(\frac{{T}_{1\mathrm{/}2}}{t}\right)}^{h}\right)}+{I}_{bottom}$$

with I_top_ and I_bottom_ being the upper and lower plateau of the current amplitude, respectively, T_1/2_ the time of half-maximal current increase, and *h* the Hill coefficient. The inflection point of the rising phase is then given by:

14 $$t_{inflection}=T_{1/2}\cdot\:\left(\frac{h-1}{h+1}\right)^\frac1h$$

### Fitting of the Goldman-Hodgkin-Katz flux equation to current/voltage curves

To determine the Na^+^ permeability (P_Na_) of oocytes expressing αβγ-ENaC under various extracellular Na^+^ concentrations, the Goldman-Hodgkin-Katz (GHK) flux equation in the form

15 $$\begin{array}{c}\:I\left(V_M,a_{Na,ex},a_{Na,in}\right)\\=P_{Na}\cdot\frac{F^2}{R\cdot T}\cdot\:V_M\cdot\:\left(\frac{a_{Na,ex}-a_{Na,in}\cdot\:e^{\frac F{R\cdot\:T}\cdot V_M}}{1-e^{\frac F{R\cdot T}\cdot\:V_M}}\right)\end{array}$$

was fitted to steady-state current/voltage (I/V) curves. Here, P_Na_ is the whole-cell Na^+^ permeability and a_Na,ex_ and a_Na,in_ the activities of Na^+^ in the external and internal solution, respectively. Since the composition of the external solution was precisely defined under all experimental conditions, a_Na,ex_ could be calculated using Eqs. 20–22, leaving only a_Na,in_ and P_Na_ as free parameters. Fitting was performed in a two-step process using the least squares method. In the first step, Eq. 15 was fitted only to the two data points located to the left and right of the reversal potential (E_rev_). This allowed for a precise estimation of E_rev_ and, due to

16 $$\:a_{Na,in}=a_{Na,ex}\cdot\:e^{-\frac F{R\cdot\:T}\cdot\:E_{rev}}$$

a good estimate of a_Na,in_. Equation 16 is a rearranged form of the Nernst equation for Na^+^. In the second step, Eq. 15 was fitted to all data points in the voltage range between V_M_ = −120 mV and − 10 mV with P_Na_ being the only free parameter.

To quantify the ENaC current run-down, the permeability at [Na^+^]_ex_ = 1 mM was determined at the start (P_Na_^start^(1 mM)) and at the end (P_Na_^end^(1 mM)) of the protocol (Fig. 6a). From these permeabilities, a time-dependent correction factor f_corr_ was calculated for each oocyte:

17 $$\:f_{corr}\left(t\right)=1-\frac{\left(P_{Na}^{start}\left(1\:mM\right)-P_{Na}^{end}\left(1\:mM\right)\right)}{P_{Na}^{start}\left(1\:mM\right)}\cdot\frac t{t_{end}}$$

where t_end_ is the time between the two voltage-step protocols which were applied at the beginning (t = 0) and at the end of the protocol in the presence of [Na^+^]_ex_ = 1 mM and in the absence of amiloride. t_end_ was 735.6 s in all recordings. The run-down-corrected Na^+^ permeabilities (P’_Na_([Na^+^]_ex_)) were then calculated as follows:

18 $$\:{P\operatorname{' }}_{Na}\left({\left[{Na}^+\right]}_{ex}\right)=\frac1{f_{corr}\left(t\right)}\cdot\:P_{Na}\left({\left[{Na}^+\right]}_{ex}\right)$$

To determine SSI for different extracellular Na^+^ concentrations, the corresponding amiloride-sensitive I/V relationships were fitted using the following modified GHK flux equation:

19 $$\begin{array}{c}I=f_{corr}(t)\cdot P_{Na}\left(1\;mM\right)\cdot\varepsilon\left({\left[Na^+\right]}_{ex}\right)\\\cdot\left(1-SSI\left({\left[Na^+\right]}_{ex}\right)\right)\cdot\frac{F^2}{R\cdot T}\cdot V_M\cdot\left(\frac{a_{Na,ex}-a_{Na,in}\cdot e^{\displaystyle\frac F{R\cdot T}\cdot V_M}}{1-e^{\displaystyle\frac F{R\cdot T}\cdot V_M}}\right)\end{array}$$

with ε([Na^+^]_ex_) being a [Na^+^]_ex_-dependent correction factor (Eq. 29) which accounts for the [Na^+^]_ex_-dependence of the open-channel Na^+^ permeability as described in the results section.

### Calculation of ion activities

The GHK flux equation (Eq. 15) is strictly valid only if the ion activities *a*_*i*_ are used instead of molar ion concentrations *c*_*i*_. The activity *a*_*i*_ of any ion species *i* in solution is given by

20 $$\:a_i={\gamma\:}_i\cdot c_i$$

with *γ*_*i*_ being the activity coefficient. To calculate the activity coefficient for Na^+^ (*γ*_*Na*_) the extended Debey-Hückel equation [6] was used:

21 $$log_{10}\left(\gamma_{Na}\right)=-\frac{A\cdot\sqrt I}{1+B\cdot r_{Na}\sqrt I}$$

with *A* = 0.509 M^− 1/2^, B = 3.28 M^− 1/2^nm^− 1^ [15] and the effective ion radius *r*_*Na*_ = 0.4 nm [30]. The ionic strength *I* was calculated from the concentration *c*_*i*_ and electric valence *z*_*i*_ of all ion species *i* in the solution according to

22 $$I=\frac12\cdot{\textstyle\sum_{}}c_i\cdot z_i^2$$

### Data analyses and statistics

The number of individual recordings is provided with *n*, numbers of oocyte donors are provided as *N*. Statistical analyses were performed with GraphPad Prism v.11.0.2 (GraphPad Software, Boston, MA, USA). The Anderson-Darling test was used to assess normal distribution of the experimental data, and appropriate parametric or non-parametric tests were used as indicated in the figure legends. Statistical significance was accepted when *p* < 0.05.

## Results

### Instrumentation-dependent inconsistencies in measured peak currents complicate the calculation of SSI from [Na^+^]_ex_-jump experiments

ENaC variants are routinely expressed in *Xenopus laevis* oocytes and characterised electrophysiologically using the two-electrode voltage-clamp (TEVC) method. To determine sodium self-inhibition (SSI), the transient response of the ENaC-mediated inward current to a rapid change in extracellular Na^+^ concentration ([Na^+^]_ex_) is usually measured at a constant membrane voltage during a TEVC experiment. We have conducted such experiments, which we refer to as [Na^+^]_ex_-jump experiments, on oocytes expressing human αβγ-ENaC using manual and automated TEVC (Fig. 1a). In both setups, the oocytes were clamped to a membrane potential (V_M_) of −60 mV and initially perfused with 1 mM Na^+^ solution (1 mM NaCl, 89 mM NMDG-Cl, 1 mM KCl, 2 mM CaCl_2_, 5 mM HEPES, pH 7.4) until a stable baseline current was achieved. The [Na^+^]_ex_-jump was then performed by switching the perfusion from 1 mM Na^+^ to 90 mM Na^+^ solution (90 mM NaCl, 1 mM KCl, 2 mM CaCl_2_, 5 mM HEPES, pH 7.4). After a stable equilibrium current was reached (1 to 3 min after the [Na^+^]_ex_-jump to 90 mM), 100 µM amiloride was washed in to completely inhibit the ENaC-mediated current. The [Na^+^]_ex_-jump from 1 mM to 90 mM led to a rapid rise in the ENaC-mediated inward Na^+^ current in both manual and automated TEVC recordings (Fig. 1a). The amplitude of the Na^+^ inward current reaches a peak value (apparent peak current, I_peak,app_) a few seconds after the [Na^+^]_ex_-jump before decaying within about 60 s to a lower equilibrium current level (apparent equilibrium current, I_eq,app_). The current decay following I_peak, app_ is caused by binding of external Na^+^ to a regulatory Na^+^-binding site in ENaC’s α-subunit [27, 40], which promotes closing of the pore gate and consequently a reduction in P_O_ (Fig. 1d). This mechanism is referred to as SSI. SSI is commonly quantified from recordings as in Fig. 1a as the fractional current decay from I_peak,app_ to I_eq,app_:

23 $$\:{SSI}_{app}=\frac{{I}_{peak,app}\mathrm{-}{I}_{eq,app}}{{I}_{peak,app}}=1-\frac{{I}_{eq,app}}{{I}_{peak,app}}$$

We define the SSI value determined in this way as apparent SSI (SSI_app_) since the calculation assumes that the P_O_ at the time of the current peak (I_peak,app_) is still equal to the P_O_ prior to the [Na^+^]_ex_-jump from 1 mM to 90 mM and that the solution exchange around the oocyte is complete.

Analysis of manual TEVC recordings as in Fig. 1a yields a SSI_app_ value of 0.37 ± 0.09 (*n* = 24) for human αβγ-ENaC (Fig. 1b). In contrast, analysis of current traces recorded using the automated TEVC system yields a significantly lower SSI_app_ value of only 0.24 ± 0.09 (*n* = 9) (Fig. 1b). This result is surprising given that, in both cases, the oocytes that were used expressed the same human ENaC subunits. Since oocytes from multiple oocyte donors were used for both sets of [Na^+^]_ex_-jump experiments (*N* = 6 and *N* = 3 for manual and automated TEVC recordings, respectively), batch-dependent differences in the post-translational processing of the expressed channels can largely be ruled out as a cause of the different SSI_app_ values.

Further analysis of the current responses recorded using the manual and automated TEVC systems reveals that they not only differ in SSI_app_, but also in terms of the time that elapsed between the onset of the current rise (rising phase) and its peak: while the time-to-peak (TTP) for manual recordings is 4.9 ± 1.3 s, the peak current in automated recordings is reached on average about 2 s later (TTP = 7.1 ± 2.6 s) (Fig. 1c). Additionally, the exponential current decay following the peak current differs drastically between both systems: In manual recordings, the current decays with an exponential time constant τ_app_ = 7.8 ± 2.3 s, which is almost twice as fast as in the automated recordings, for which an average time constant of τ_app_ = 14.3 ± 7.5 s can be determined (Supplementary Figure  S1 a). Further analysis also shows that the reciprocal τ_app_ values (1/τ_app_) exhibit a strong negative and positive correlation with the corresponding TTP and SSI_app_ values, respectively (Supplementary Figure  S1 b, c), suggesting that all three parameters (TTP, SSI_app_, τ_app_) are affected by the same confounding factor. This confounding factor is very likely the perfusion rate.

These findings suggest that the increase in [Na^+^]_ex_ occurs faster in the manual compared to the automated TEVC system. In the automated system, perfusion is achieved using a peristaltic pump. For the [Na^+^]_ex_-jump experiments, an optimised rotation speed was used, resulting in a flow rate of ~ 5 ml/min. In the manual TEVC recordings, bath solution exchange was performed using a gravity-based perfusion system, which allowed a 4 times faster flow rate of ~ 20 ml/min.

The differences in the measured TTP, SSI_app_ and τ_app_ values are probably not only caused by the different flow rates, but also by significant differences in the geometries of the perfusion chambers used. For manual recordings, the oocyte is placed in the middle of an elongated chamber with the inlet and outlet located at the opposite ends (Fig. 1e). Due to this channel-like design, the inflowing solution physically pushes the solution in the chamber toward the outlet resulting in a convection-dominated fluid flux which allows a fast and complete solution exchange around the oocyte. The automated TEVC system, on the other hand, uses standard 96-well plates with V-shaped wells (Fig. 1f). The oocytes settle at the bottom of the wells and can be impaled automatically for TEVC recordings by a robotic arm carrying both the glass microelectrodes and the reference electrodes, as well as the inlet and outlet tubing. This design, which makes the automation and use of 96-well plates possible in the first place, causes the solution exchange to occur primarily through mixing and dilution, resulting in gradual rather than stepwise concentration changes. The differences in the design of the perfusion chambers and flow rates suggest that the change in [Na^+^]_ex_ around the oocyte following a [Na^+^]_ex_-jump occurs more slowly in the automated TEVC system than in the manual one. However, is this difference a sufficient explanation for the differing SSI_app_ values obtained for human αβγ-ENaC? To answer this question, we decided to investigate the influence of the perfusion rate on the current responses during [Na^+^]_ex_-jump experiments using numerical simulations.

### SSI values obtained from [Na^+^]_ex_-jump experiments are highly dependent on the perfusion rate

As postulated earlier, the [Na^+^]_ex_-dependent gating, i.e. SSI, of ENaC can be mathematically described using a simple two-state Markov model [14]. This model (Fig. 2a_**(i)**_) assumes that ENaC can exist in two conformational states, an active (A) and an inhibited state (I). The transitions between these two states are determined by the inhibition (*k*_*AI*_) and activation (*k*_*IA*_) rate constants with *k*_*AI*_ depending linearly on [Na^+^]_ex_:

24 $$k_{AI}=k'_{AI}\cdot{\left[Na^+\right]}_{ex}$$

with *k’*_*AI*_ being the inhibition rate constant at [Na^+^]_ex_ = 1 mM. The linear [Na^+^]_ex_-dependence of *k*_*AI*_ is the direct consequence of the binding of a single Na^+^ ion to the Na^+^ binding site (Na^+^-BS) of the α-subunit (Fig. 2a_(ii)_). The model assumes that the binding of a Na^+^ ion to the Na^+^-BS leads without any time delay to the closing of the pore and, consequently, transition to the inhibited state I. Conversely, it is assumed that the dissociation of the Na^+^ ion from the Na^+^-BS immediately leads to the transition to the active state A. It is further assumed that in the active state, the pore is Na^+^ conductive, whereas in the inhibited state, the pore is fully closed and thus non-conductive (Fig. 2a_(ii)_). The voltage- and [Na^+^]-dependence of the Na^+^ current through the channel in the active state is modelled using the Goldman-Hodgkin-Katz (GHK) flux model (Eq. 6).

It should be noted that the gating model represents a simplification of the conformational changes occurring in the channel protein during SSI. However, this model is sufficiently complex to reproduce the time- and [Na^+^]_ex_-dependence of the ENaC-mediated Na^+^ current observed in a [Na^+^]_ex_-jump experiment.

We therefore used this model to simulate [Na^+^]_ex_-jump experiments similar to those shown in Fig. 1a. In these simulations, V_M_ was set to −60 mV and the internal Na^+^ concentration ([Na^+^]_in_) was assumed to be 5 mM. The only parameter changed during a simulation was the external Na^+^ concentration: During the first 30 s, [Na^+^]_ex_ was set to 1 mM. The [Na^+^]_ex_-jump was then simulated by increasing the Na^+^ concentration of the perfused external solution to 90 mM. Finally, [Na^+^]_ex_ was reduced back to 1 mM.

If the external solution is replaced instantaneously, i.e. with an infinitely high perfusion rate (D = ∞), the current response can be calculated analytically (Eqs. 1, 5 and 6). If the rate constants are chosen such that the sodium self-inhibition at [Na^+^]_ex_ = 90 mM is SSI_true_ = 0.5 and the exponential time constant of the current decay following the [Na^+^]_ex_-jump to 90 mM is τ_true_ = 10 s, the model yields the current response (normalised to the equilibrium current at [Na^+^]_ex_ = 90 mM) shown in blue in Fig. 2b. In this case, the highest current (I_peak,true_) occurs immediately after the [Na^+^]_ex_-jump to 90 mM at I_norm_ = −2. This is because the probability that the channels are in the Na^+^-conductive state A (P_A_) does not change abruptly with the change in [Na^+^]_ex_, but approaches the new equilibrium value P_A,eq_ exponentially with the time constant τ_true_ as described by Eq. 1. In contrast to P_A_, the current conducted by an active channel (Eq. 6) responds instantly to the sudden change in the [Na^+^]-gradient; therefore, the time course of the current response following the [Na^+^]_ex_-jump (Fig. 2b, blue trace) mirrors the time-dependent change in P_A_. Here, I_peak,true_ corresponds to the P_A_ value immediately before the [Na^+^]_ex_-jump of P_A,eq_(1 mM) ≈ 1, and the equilibrium current (I_eq,true_) at I_norm_ = −1 to the channel activity reduced by SSI to P_A,eq_ (90 mM) = 0.5. The current decays exponentially from I_peak,true_ to I_eq,true_ with the time constant τ_true_ = 10 s.

In an electrophysiological experiment, however, the change in [Na^+^]_ex_ does not occur instantaneously, but in a time-dependent manner due to mixing effects in the recording chamber. To account for this time-dependent change in [Na^+^]_ex_ following a [Na^+^]_ex_-jump, we treat the recording chamber in our numerical simulations as ideal continuous stirred tank reactor (ICSTR). In short, this model assumes that the volume of the external solution in the recoding chamber remains constant during perfusion (influx rate = efflux rate) and that mixing within the chamber occurs instantaneously. This model enables the simulation of different solution exchange speeds by varying the perfusion rate D which is defined as the number of reactor volumes passing through the recording chamber per unit of time. In case of the automated TEVC system, the volume in the recording chamber was approximately V = 200 µl and the flow rate was Q ≈ 5 ml/min. Under ideal mixing conditions in the recording chamber, as assumed in the ICSTR model, the perfusion rate D of the automated TEVC system should therefore be D = Q/V = 0.42 s⁻¹. However, since the ICSTR model is a simplification of the actual conditions in the reaction chamber, D does not necessarily correspond to the influx and efflux rates of the perfusion system. Within the recording chamber there may be areas with different local perfusion rates. Particularly with a chamber design such as that shown in Fig. 1f, it is likely that the solution exchange occurs more rapidly in the upper region of the chamber than in the lower one, with the result that the local perfusion rate in the vicinity of the oocyte may be lower than the influx and efflux rates. Therefore, D may be considered as a local perfusion rate in the vicinity of the oocyte. To determine the actual perfusion rate D experienced by an oocyte in the automated TEVC system, we analysed the rising phase of ENaC-mediated transient currents from [Na^+^]_ex_-jump experiments as shown in Fig. 1a under conditions in which Na^+^ self-inhibition has been abolished (Supplementary Figure S6) (for details see Appendix S4). This analysis yielded a perfusion rate of D = 0.19 ± 0.11 s^− 1^ (*n* = 5) (Supplementary Figure  S1 d). The actual perfusion rate in the vicinity of the oocyte is therefore only about half of the value calculated under the assumption of ideal mixing conditions. The analysis of similar experiments performed with the manual TEVC system revealed that the solution exchange in the vicinity of oocytes during manual TEVC recordings occurs with a perfusion rate of 0.33 ± 0.06 s^− 1^ (*n* = 8). The mean perfusion rate of the manual TEVC system is therefore approximately 70% higher than the perfusion rate in the automated TEVC system (Supplementary Figure  S1 d). Close inspection of ENaC-mediated transient currents reported in other studies show steeper rising phases in some cases (e.g. Chraïbi and Horisberger [14],, suggesting that higher perfusion rates can be achieved by optimising the design of the recording chamber. Therefore, for the following investigations we define the range between D = 0.1 s⁻¹ and 1 s⁻¹ as experimentally feasible.

We therefore decided to investigate the effect of the time-dependent solution exchange on ENaC-mediated currents for D values between 0.05 s⁻¹ and 10 s⁻¹. As illustrated by the simulated current traces in Fig. 2b, reducing the perfusion rate from D = 10 s^− 1^ to 0.05 s^− 1^ results in a continuous and strong decrease of I_peak,app_. Plotting the I_peak,app_ normalised to the absolute value of the equilibrium current (|I_eq_|) against the corresponding perfusion rate (Fig. 2c) reveals that I_peak,app_ decreases almost exponentially as the perfusion rate slows down. Lowering the perfusion rate to D = 1 s⁻¹, which we consider to be close to the maximal perfusion rate in our manual TEVC system, already leads to a decrease in the normalised peak current from − 2 to −1.7, which corresponds to a loss in peak-to-equilibrium amplitude (= I_peak,true_-I_eq,true_) of 30%. In the experimentally feasible range between D = 1 s⁻¹ and 0.1 s⁻¹, the normalised peak current further drops to −1.19, i.e. to only 19% of the true peak-to-equilibrium amplitude. This strong effect of the perfusion rate on I_peak, app_ is reflected in a correspondingly high sensitivity of SSI_app_ to changes in the perfusion rate: A reduction from D = 1 s⁻¹ to 0.1 s⁻¹ results in a decrease in SSI_app_ from 0.42 to 0.15 (Fig. 2d), which corresponds to an underestimation of the true SSI value by 16% to 70%.

In addition, we analysed the current decay following I_peak,app_ by fitting the exponential part of the falling phase with a single exponential function (Eq. 11). This yields the apparent time constant τ_app_ shown in Fig. 2e. Interestingly, τ_app_ is identical to τ_true_ over a wide range of perfusion rates. Only below approximately 0.5 s⁻¹, τ_app_ increases abruptly. This is due to the fact that for D < 0.5 s⁻¹, the time constant of [Na⁺]_ex_ exchange (τ_exchange_ = 1/D) reaches a value similar to τ_true_ and eventually exceeds it. This means that, for very slow perfusion rates, the increase in [Na^+^]_ex_ occurs so slowly that P_A_ is close to equilibrium at any time, resulting in the disappearance of a current peak. However, Fig. 2e illustrates that τ_app_ is a good estimate of τ_true_ – and thus the kinetics of [Na^+^]_ex_-dependent gating – even within the range of experimentally feasible perfusion rates.

In summary, the results of these simulations show that the differences in solution exchange kinetics between the manual and automated TEVC systems can sufficiently explain the discrepancy between the SSI_app_ values measured for αβγ-ENaC using these systems. In addition, these simulations reveal that SSI_app_ deviates significantly from SSI_true_ even at high perfusion rates and is thus not suitable as a reliable measure of SSI in ENaC variants.

### Estimation of the true peak current in [Na^+^]_ex_-jump experiments by backward extrapolation

**Fig. 3:**
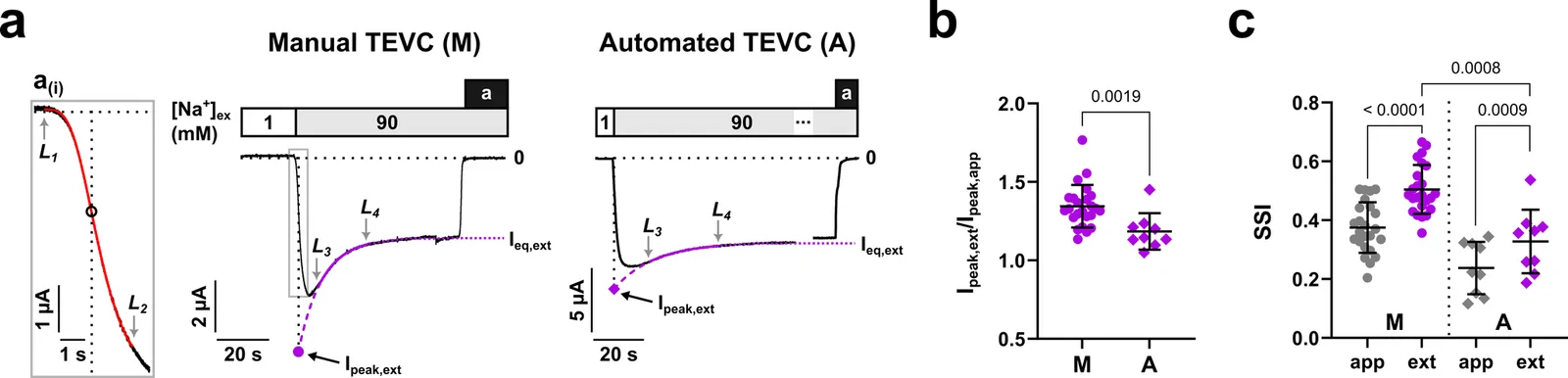
Application of the peak-extrapolation method to current responses of oocytes expressing αβγ-ENaC from [Na^+^]_ex_-jump experiments performed using manual and automated TEVC. (**a**) Application of the peak-extrapolation method to the current traces shown in Fig. 1a. The rising phase of the current trace recorded with the manual TEVC is highlighted by a grey rectangle and shown enlarged in (**a**_(i)_). Application of the peak extrapolation method comprises two fitting steps: (1) Fitting of the modified Hill equation (Eq. 13) to the current between time points *L*_*1*_ and *L*_*2*_ of the rising phase (red line in (**a**_(i)_)) yields the inflection point of the rising phase (black open circle in (**a**_(i)_)), which is defined as t = 0. (2) Fitting of a single exponential function (solid purple line, Eq. 11) to the current between time points *L*_*3*_ and *L*_*4*_ of the falling phase yields both the equilibrium current I_eq,ext_ and, via backward extrapolation (dashed purple line), the extrapolated peak current I_peak,ext_ at t = 0. (**b**) Ratio of extrapolated peak current I_peak,ext_ to corresponding apparent peak current I_peak,app_ from manual (M) and automated (A) modes. Statistical analysis was performed using Mann-Whitney U-test. (**c**) Comparison of apparent SSI values (app) and SSI values obtained by applying the peak-extrapolation method (ext) to current traces as in (a). Statistical comparisons of apparent (app) and corresponding extrapolated (ext) SSI values were performed using Student’s paired t-tests. Statistical comparison between extrapolated SSI values obtained from manual (M) and automated (A) TEVC recordings was performed using a Student’s unpaired t-test with Welch’s correction. Data points in (b-d) represent data from n individual experiments and N oocyte donors; lines and error bars represent arithmetic mean ± SD. Panels (**b**–**d**), M: *n* = 24, *N* = 6; A: *n* = 9, *N* = 3

The results from the above raise the question of how SSI can be reliably determined from [Na^+^]_ex_-jump experiments, especially when systems with a limited perfusion rate are employed. The current simulations show that a reduction of the perfusion rate primarily distorts the peak current, whereas a significant distortion of the exponential current decay during the falling phase only occurs for perfusion rates < 0.5 s⁻¹. Depending on the perfusion rate, it should therefore be possible to estimate the true peak current by backward extrapolation of the exponential current decay to the beginning of the [Na^+^]_ex_-jump (t = 0) as already proposed by Chraïbi and Horisberger [14]. Since the [Na^+^]_ex_-jump does not occur instantaneously, t = 0 is set to the inflection point of the rising phase which is determined by fitting the modified Hill equation (Eq. 13) to the rising current between time points *L*_*1*_ and *L*_*2*_ as shown in Fig. 3a_(i)_. Next, the current of the falling phase is fitted with a single exponential function (Eq. 11) between the time points *L*_*3*_ and *L*_*4*_ as shown in Fig. 3a. *L*_*3*_ corresponds to the inflection point of the falling phase, while *L*_*4*_ corresponds to the time at which about 80% of the current decay had occurred. Backward extrapolation of the exponential function to the inflection point of the rising phase then yields the extrapolated peak current I_peak, ext_. Additionally, the fit provides the equilibrium current I_eq, ext_.

We have applied this extrapolation method to the current traces recorded from oocytes expressing human αβγ-ENaC using the manual and automated TEVC systems shown in Fig. 1 and subsequently calculated the ratio between the extrapolated and the corresponding apparent peak current (I_peak,ext_/I_peak,app_) for all individual measurements. For the manual recordings, this analysis reveals that I_peak,ext_ is 1.34 ± 0.14 times larger than I_peak,app_ (Fig. 3b). The same analysis yields a slightly but significantly lower ratio of I_peak,ext_/I_peak,app_ = 1.18 ± 0.12 (*p* = 0.0019) for the current traces recorded with the automated system. In contrast, the ratios between the extrapolated and the corresponding apparent equilibrium current (I_eq, ext_/I_eq, app_) are virtually identical with 1.06 ± 0.06 (*n* = 24) and 1.04 ± 0.05 (*n* = 9, *p* = 0.3182) for manual (M) and automated (A) recordings, respectively. That the I_eq,ext_/I_eq,app_ ratios are not exactly 1 is likely due to a “contamination” of I_peak,app_ by a current run-down, causing I_peak,app_ to be slightly lower than the corresponding I_eq,ext_. The impact of a potential current run-down on I_eq, ext_ was minimised by setting the right boundary (*L*_*4*_) for fitting of the falling phase with Eq. 11 to a time point at which the current is still visibly declining. If *L*_*4*_ is set to a later time point, Eq. 11 is no longer sufficient to adequately describe the current decay (Supplementary Figure S2a_(2)_) and must therefore be extended by a run-down term (Eq. 12). In fact, taking the current run-down into account has only a minor effect on I_peak,ext_, I_eq,ext_ and SSI_ext_ (Supplementary Figure S2a_(3)_,b-d).

Next, analogous to the calculation of the SSI_app_ (Eq. 23), we calculated the extrapolated SSI (SSI_ext_) from the I_peak,ext_ and I_eq,ext_ values determined using the extrapolation method:

25 $$\:{SSI}_{ext}=1-\frac{{I}_{eq,ext}}{{I}_{peak,ext}}$$

Comparison between SSI_app_ and SSI_ext_ reveals that the mean SSI_ext_ value is ~ 0.1 higher than the corresponding SSI_app_ value for both TEVC systems (Fig. 3c). However, the SSI_ext_ values obtained from manual (SSI_ext_ = 0.50 ± 0.08) and automated recordings (SSI_ext_ = 0.33 ± 0.11) still differ significantly (*p* = 0.0008). This discrepancy is likely explained by the lower perfusion rate of the automated system, which causes not only a pronounced distortion of the peak current, but also of the exponential current decay (Supplementary Figure  S1 a).

These results suggest that SSI_ext_ is a good estimate of SSI_true_ only if the time constant of the solution exchange (τ_exchange_) is significantly larger than the true time constant (τ_true_) of the current decay.

### Evaluation of the reliability of the peak-extrapolation method

**Fig. 4:**
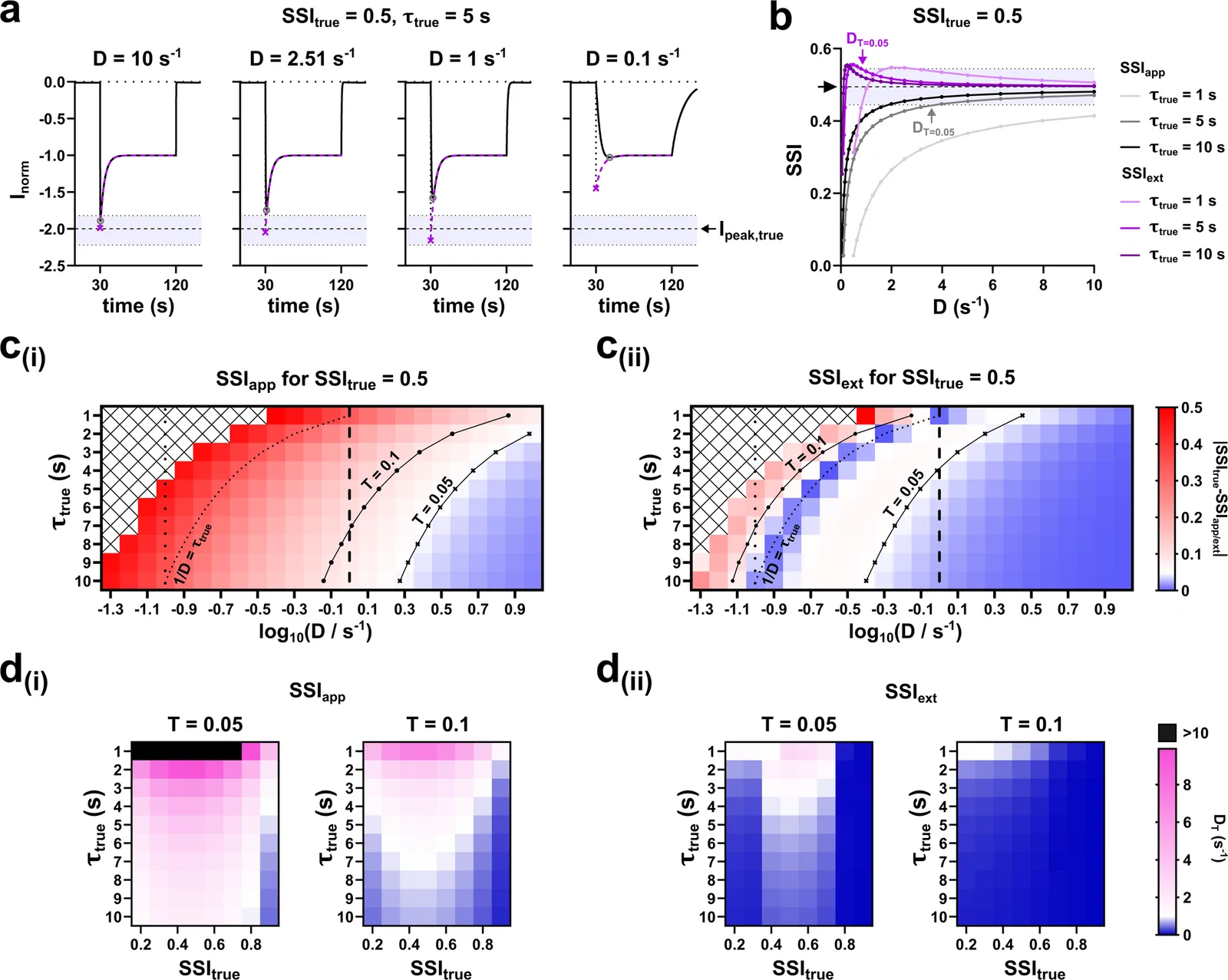
Evaluation of the reliability of the peak-extrapolation method by application to current traces simulated for different gating kinetics and perfusion rates.(**a**) Numerically simulated normalised ENaC-mediated current responses at V_M_ = −60 mV. Currents were normalised to the equilibrium current at [Na^+^]_ex_ = 90 mM. Simulations were performed as in Fig. 2 for SSI_true_ = 0.5, τ_true_ = 5 s, and different perfusion rates D as indicated above the traces. The peak-extrapolation method was applied to each simulated current trace as shown in Fig. 3a. The apparent and extrapolated current peaks are highlighted for each trace with a grey open circle and a purple cross, respectively. The dashed purple lines show the best fit of the single exponential function (Eq. 11) to the exponentially decaying part of the falling phase and the backward extrapolation to t = 0. Timepoint t = 0 was identified as shown in Fig. 2a by fitting the modified Hill equation (Eq. 13) to the rising phase of the current trace and subsequent calculation of the inflection point. The horizontal dashed line at I_norm_ = −2 indicates peak current for D = ∞ (true value). The area highlighted in light blue indicates the current range into which the apparent (I_peak,app_) or extrapolated peak current (I_peak,ext_) must fall in order for the calculated SSI value (SSI_app_ or SSI_ext_) to be SSI_true_ ± 5% (= 0.5 ± 0.05). (**b**) Perfusion-rate dependence of SSI_app_ (shades of grey) and SSI_ext_ (shades of purple) obtained from numerical simulations as in (a) for SSI_true_ = 0.5 and τ_true_ = 1 s, 5 s, and 10 s. Connecting lines between the data points (closed circles) represent linear interpolations. SSI_true_ = 0.5 is indicated by a black arrow and a horizontal dashed line. The area highlighted in light blue indicates SSI_true_ ± 0.05 (= 0.5 ± 0.05). The D-values at which the SSI_app_ and SSI_ext_ curves for τ_true_ = 5 s first exceed a deviation of T = 0.05 from SSI_true_ are indicated with D_T=0.05_ and an arrow in the corresponding colour. **(c)** Heatmaps depicting the absolute value of the deviation of SSI_app_ (**c**_**(i)**_) and SSI_ext_ (**c**_**(ii)**_) from SSI_true_ = 0.5 (|SSI_true_-SSI_app_| and |SSI_true_-SSI_ext_|, respectively) for different perfusion rates D and τ_true_ values. Each row corresponds to a curve as in (b). Since D was varied exponentially for numerical simulations, the x-axis shows the common logarithm of D. The range of perfusion rates typically used in TEVC recordings is indicated by a vertical dotted line (lower limit: 0.1 s^− 1^) and a vertical dashed line (upper limit: 1 s^− 1^). Perfusion rates D_T=0.05_ and D_T=0.1_ at which the deviation |SSI_true_-SSI_app_| or |SSI_true_-SSI_ext_| for a given τ_true_ value first exceeds the threshold T = 0.05 and T = 0.1 are plotted as connected black crosses and connected black circles, respectively. The curved dotted line marks those D values for which the time constant of the solution exchange (τ_exchange_ = 1/D) is identical to τ_true_. Colour code: |SSI_true_-SSI_app/ext_| < 0.05, shades of blue; |SSI_true_-SSI_app/ext_| = 0.05, white; |SSI_true_-SSI_app/ext_| > 0.05, shades of red. Fields containing a black cross correspond to τ_true_/D combinations that did not result in a peak in the simulated current trace. (**d**) Heatmaps depicting the highest perfusion rate (D_T_) at which |SSI_true_-SSI_app_| (**d**_**(i)**_) and |SSI_true_-SSI_ext_| (**d**_**(ii)**_) exceeds T = 0.05 (left) and T = 0.1 (right). D_T_ values were calculated from curves as in (b) for SSI_true_ = 0.2 to 0.9 and τ_true_ = 1 s to 10 s. Colour code: D_T_ < 1 s^− 1^, shades of blue; D_T_ = 1 s^− 1^, white; D_T_ > 1 s^− 1^, shades of pink; D_T_ > 10 s^− 1^, black

To assess the reliability of SSI_ext_ as an estimate of SSI_true_, we decided to evaluate the extrapolation method using numerically simulated [Na^+^]_ex_-jump experiments as in Fig. 2. To simulate ENaC variants with different gating kinetics, SSI_true_ and τ_true_ were systematically varied between 0.2 and 0.9, and between 1 s and 10 s, respectively, resulting in 80 different SSI_true_/τ_true_ combinations. For each SSI_true_/τ_true_ combination, [Na^+^]_ex_-jump experiments were simulated for perfusion rates ranging from *D* = 0.01 to 10 s⁻¹ and automatically analysed using the extrapolation method. Exemplary current traces simulated for SSI_true_ = 0.5 and τ_true_ = 5 s are shown in Fig. 4a. For this SSI_true_/τ_true_ combination, at D = 2.51 s^− 1^ I_peak, app_ has already decreased to such an extent that the resulting SSI_app_ value deviates by more than 0.05 from SSI_true_, as indicated by I_peak, app_ lying outside the area highlighted in light blue. Closer examination of the relationship between SSI_app_ and D for this SSI_true_/τ_true_ combination (Fig. 4b) reveals that a deviation of 0.05 from SSI_true_ is already exceeded at D = 3.8 s⁻¹. In the following, we refer to the perfusion rate, at which the deviation of SSI_app_ or SSI_ext_ from SSI_true_ (|SSI_true_-SSI_app_| or |SSI_true_-SSI_ext_|) exceeds the threshold T = 0.05 for the first time, as D_T=0.05_.

The analysed current traces shown in Fig. 4a suggest that, within the range of examined perfusion rates, the extrapolated peak current I_peak,ext_ is closer to I_peak,true_ than the apparent peak current I_peak,app_. Consequently, SSI_ext_ should always be closer to SSI_true_ than the corresponding SSI_app_ value. This is confirmed by comparing the SSI_ext_/D and SSI_app_/D relationships in Fig. 4b: for all three τ_true_ values (10 s, 5 s and 1 s), the SSI_ext_ curve leaves the SSI_true_ ± 0.05 zone at significantly lower D_T=0.05_ values than the corresponding SSI_app_ curve. For example, the D_T=0.05_ value derived from the SSI_ext_ curve for τ_true_ = 5 s is with 0.8 s^− 1^ almost five times smaller than D_T=0.05_ = 3.8 s^− 1^ determined from the corresponding SSI_app_ curve. A major difference between the SSI_app_ and SSI_ext_ values is that the former are consistently below SSI_true_ for all D values, whereas the SSI_ext_ values initially rise above SSI_true_ as the perfusion rate decreases and only fall below SSI_true_ and towards zero at very low perfusion rates. As a result, the SSI_ext_ curves of the three SSI_true_/τ_true_ combinations shown in Fig. 4b first exceed the threshold T = 0.05 at SSI_ext_ = 0.55, while the corresponding SSI_app_ curves first exceed this threshold at SSI_app_ = 0.45. Hence, while SSI_app_ always underestimates SSI_true_, SSI_ext_ can either be an over- or an underestimation of SSI_true_ depending on the perfusion rate.

To assess the extent to which the extrapolation method improves the determination of SSI for different ENaC variants, we calculated the deviations of the SSI_app_ and SSI_ext_ values from SSI_true_ for all 80 SSI_true_/τ_true_ combinations as a function of the perfusion rate. The heatmaps provided in Fig. 4c show these deviations in colour-coded form for SSI_true_ = 0.5 and τ_true_ values ranging from 1 s to 10 s (i.e. for 10 SSI_true_/τ_true_ combinations). For SSI_app_ (Fig. 4c_(i)_), it is evident that the deviation from SSI_true_ increases when either τ_true_ of the ENaC variant or D decreases. This result, which was found for all examined SSI_true_ values (Supplementary Figure S3), does not come as a surprise, as I_peak,app_ must decrease when either the channel’s response to a change in [Na^+^]_ex_ becomes faster or the solution exchange slows down. Remarkably, SSI_app_ deviates for the majority of all SSI_true_/τ_true_ combinations by more than 0.1 from SSI_true_ across the range of experimentally feasible perfusion rates (Fig. 4c_(i)_ and Supplementary Figure S3). This result is further reflected by the observation that D_T=0.05_ and D_T=0.1_ are exceeding 1 s⁻¹ for about 90% and about 50% of all SSI_true_/τ_true_ combinations (Fig. 4d_**(i)**_), respectively, confirming that SSI_app_ is not suitable as a reliable estimate of SSI_true_.

In contrast, Fig. 4c_**(ii)**_ shows that the deviation of SSI_ext_ from SSI_true_ = 0.5 is consistently less than 0.1 for all simulations in which the time constant of solution exchange (τ_exchange_ = 1/D) is smaller than τ_true_. The same result is found for all simulations with SSI_true_ ≥ 0.4 (Supplementary Figure S3). Interestingly, for SSI_true_ < 0.4 and τ_true_ = 1 s to 10 s, the corresponding D_T=0.05_ and D_T=0.1_ values are almost identical and lie only slightly above the perfusion rate at which τ_exchange_ is equal to τ_true_ (Supplementary Figure S3). This means that for SSI_true_ < 0.4, the deviation |SSI_true_-SSI_ext_| remains between 0 and 0.05 for almost all perfusion rates larger than the critical value of D = 1/τ_true_, and only rises abruptly when D closely approaches this critical value. Therefore, this analysis reveals that SSI_ext_ represents a good estimate of SSI_true_ as long as the perfusion rate D is larger than 1/τ_true_. Notably, for ENaC variants with SSI_true_ > 0.6, SSI_ext_ remains a good estimate even when D is substantially lower than 1/τ_true_ (Supplementary Figure S3). That SSI_ext_ provides a better estimate of SSI_true_ than SSI_app_ for all SSI_true_/τ_true_ combinations examined is also evident from a comparison of the heat maps shown in Fig. 4d: While D_T=0.05_ for SSI_app_ lies within the range of experimentally feasible perfusion rates for only 9% of all SSI_true_/τ_true_ combinations (Fig. 4d_**(i)**_), this is the case for 85% of the combinations after applying the extrapolation method (Fig. 4d_**(ii)**_). D_T=0.1_ is even below 1 s⁻¹ for 99% (i.e. 79 out of 80) of all SSI_true_/τ_true_ combinations after applying the extrapolation method (Fig. 4d_**(ii)**_).

In summary, SSI_ext_ values obtained using the extrapolation method generally provide a good estimate of SSI_true_ (i.e. deviating from SSI_true_ by less than 0.1), provided that τ_exchange_ > τ_true_ is fulfilled. However, the extrapolation method fails once τ_true_ ≈ τ_exchange_, as this results not only in a substantial distortion of the current peak, but also of the exponential current decay (Supplementary Figure S4). Naturally, τ_true_ of a given ENaC variant is not known a priori, and it is therefore not immediately apparent whether the perfusion rate used is sufficiently high to exclude distortion of the exponential phase. It would therefore be desirable to have a method available that allows determination of SSI independently of the perfusion rate.

### The β-subunit S520C mutant allows determination of SSI by chemical modification in a perfusion-independent manner

A well-established method for determining activity of ENaC variants is the introduction of a cysteine into the conserved degenerin site at amino acid position 520 of the human β-subunit, followed by covalent modification of the introduced cysteine residue with the sulfhydryl reactive compound 2-(trimethylammonium)ethyl methanethiosulfonate (MTSET) [57]. The covalent binding of MTSET to β-Cys_520_ causes human αβ_S520C_γ-ENaCs to become locked in the active state, thereby increasing the probability to find the channel in the active state to P_A_ ≈ 1 [57]. Assuming that the MTSET modification does not alter the open-channel current amplitude in human ENaCs, P_A_ can be estimated by comparing the ENaC-mediated currents before (I_eq,1_) and after MTSET coupling (I_eq,2_), assuming that the number of channels has not changed:

26 $$\:{P}_{A\:}\text{}\mathrm{=}\:\text{}\frac{{I}_{eq,1}}{{I}_{eq,2}}$$

This method has been previously used to estimate the open probability of human [20, 57], rat [29], guinea pig [34] and *Xenopus laevis* [63] ENaCs. SSI can then be estimated from P_A_ as follows:

27 $$\:{SSI}_{MTSET\:}\text{}\mathrm{=}\:\text{}1-{P}_{A}$$

**Fig. 5:**
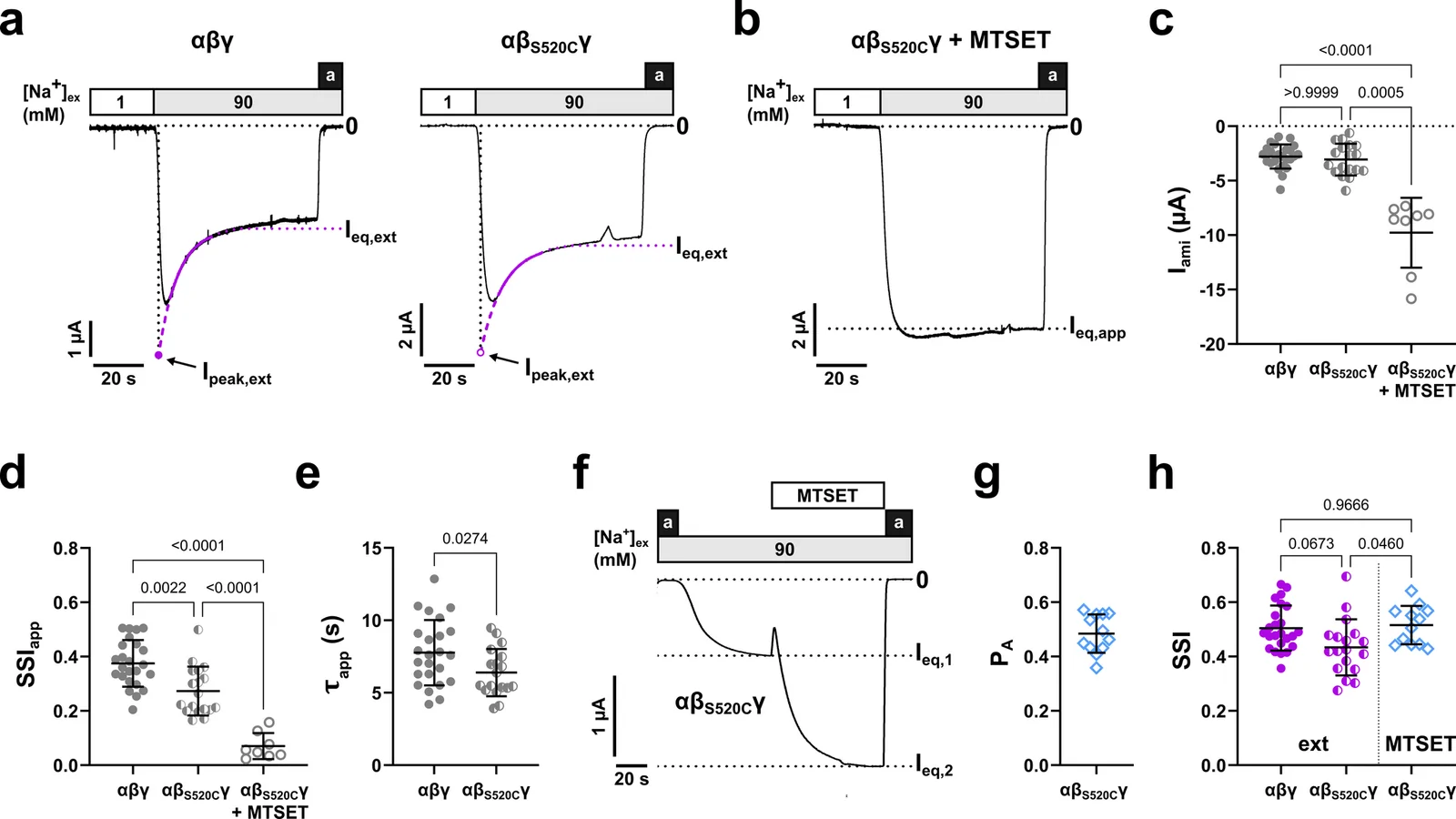
Estimation of steady-state SSI by chemical modification of αβ_S520__C_γ-ENaC with MTSET.(**a**) Representative transmembrane current (I_M_) traces from manual TEVC recordings at V_M_ = −60 mV of oocytes expressing wild-type αβγ-ENaC (left) and the MTSET-sensitive αβ_S520C_γ-ENaC mutant (right). [Na^+^]_ex_ was changed from 1 mM to 90 mM; 100 µM amiloride (a; black box) was applied at the end of each recording. Solid purple lines show the best fit of the single exponential function (Eq. 11) to the exponentially decaying part of the falling phase. Dashed purple lines show the backward extrapolation to t = 0. t = 0 was identified by fitting the modified Hill equation (Eq. 13) to the rising phase of the current trace and subsequent calculation of the inflection point. The extrapolated current peaks are highlighted as purple circles. Dotted purple lines show the forward extrapolation yielding the equilibrium current level I_eq,ext_. (**b**) Representative I_M_ trace from a manual TEVC recording as in (a) of an oocyte expressing αβ_S520C_γ-ENaC after 5 min treatment with 1 mM MTSET in 1 mM Na^+^ solution. The apparent equilibrium current (I_eq,app_) was measured 60 s after the [Na^+^]_ex_-jump to 90 mM. **(c)** Amiloride-sensitive currents (I_ami_) calculated from TEVC recordings as in (a) and (b) performed on oocytes expressing wild-type αβγ-ENaC (*n* = 24, *N* = 6) and αβ_S520C_γ-ENaC without (*n* = 18, *N* = 4) and after treatment with 1 mM MTSET (+ MTSET; *n* = 8, *N* = 3). I_ami_ was calculated as the difference between I_eq,app_ and the steady-state current measured in the presence of 100 µM amiloride. Statistical analysis was performed using a Kruskal-Wallis test with Dunn’s multiple comparisons test. (**d**) Apparent sodium self-inhibition (SSI_app_) of αβγ-ENaC (*n* = 24, *N* = 6) and αβ_S520C_γ-ENaC without (*n* = 18, *N* = 4), and after treatment with 1 mM MTSET (+ MTSET; *n* = 8, *N* = 3). SSI_app_ values were calculated from I_peak,app_ and I_eq,app_ obtained from current traces as in (a) and (b). Statistical analysis was performed using a Brown-Forsythe and Welch with Dunnett´s T3 multiple comparisons test. (**e**) Apparent time constants (τ_app_) of the current decay following the [Na^+^]_ex_-jump to 90 mM from oocytes expressing αβγ-ENaC (*n* = 24, *N* = 6) and αβ_S520C_γ-ENaC (*n* = 18, *N* = 4). Individual τ_app_ values were determined by fitting a single exponential function (Eq. 11) to the exponentially decaying part of the falling phase. Statistical analysis was performed using Student’s unpaired t-test with Welch’s correction. (**f**) Representative transmembrane current (I_M_) trace from an automated TEVC recording at V_M_ = −60 mV of an oocyte expressing MTSET-sensitive αβ_S520C_γ-ENaC. Equilibrium currents in [Na^+^]_ex_ = 90 mM were measured in the absence (I_eq,1_) and presence of 1 mM MTSET (I_eq,2_). 100 µM amiloride were applied at the beginning and end of each recording (a; black boxes). (**g**) Estimated active-state probability (P_A_) of αβ_S520C_γ-ENaC calculated as P_A_ = I_eq,1_/I_eq,2_ from recordings as in (f) (*n* = 12, *N* = 3). (**h**) Comparison of SSI values obtained by applying the peak-extrapolation method to manual TEVC recordings as in (a) (ext) and SSI values determined from automated MTSET experiments as in (g) (MTSET). Statistical analysis was performed using Brown-Forsythe and Welch ANOVA tests with Dunnett’s T3 multiple comparisons test. Data points in (c-e) and (g, h) represent data from n individual experiments; lines and error bars represent arithmetic mean ± SD

To determine the SSI_MTSET_ for αβγ-ENaC, we introduced the S520C mutation into the human β-subunit and expressed it together with the human α- and γ-subunits in *X. laevis* oocytes. First, we demonstrated with [Na^+^]_ex_-jump experiments (Fig. 5a, b) that pretreatment of αβ_S520C_γ-ENaC-expressing oocytes with 1 mM MTSET for 5 min results in the reduction of SSI_app_ to a value close to zero (SSI_app_ = 0.07 ± 0.05, *n* = 8) (Fig. 5d) and to a 3.2-fold increase in the amiloride-sensitive equilibrium current compared with untreated oocytes (Fig. 5c). This result confirms that binding of MTSET to Cys_520_ in the β_S520C_ subunit suppresses the transition of the channel into the Na^+^-inhibited state. Additionally, comparison of the amiloride-sensitive equilibrium currents in oocytes expressing αβγ- (I_ami_ = −2.8 ± 1.1 µA) and αβ_S520C_γ-ENaC (I_ami_ = −3.1 ± 1.4 µA) shows that the mutation has no significant effect on equilibrium channel activity (Fig. 5c).

To calculate P_A_ of αβ_S520C_γ-ENaC, we next performed experiments in which we recorded αβ_S520C_γ-ENaC-mediated Na⁺ currents at V_M_ = −60 mV in the presence of 90 mM external Na^+^ before and after MTSET treatment in the same oocyte using the automated TEVC system (Fig. 5f). From the equilibrium currents I_eq,1_ and I_eq,2_ recorded in *n* = 12 such experiments, we estimated an active-state probability of P_A_ = 0.48 ± 0.07, which, according to Eq. 27, corresponds to a Na^+^-dependent inhibition of SSI_MTSET_ = 0.52 ± 0.07 (Fig. 5h). This value is slightly higher than the SSI_ext_ value of the mutant channel αβ_S520C_γ-ENaC (SSI_ext_ = 0.43 ± 0.10) and almost identical to the SSI_ext_ value of wildtype αβγ-ENaC (SSI_ext_ = 0.50 ± 0.08).

From these results we conclude that, in case of human αβγ-ENaC, the perfusion rate-dependent [Na^+^]_ex_-jump experiments can be replaced by the perfusion rate-independent MTSET experiments. However, as both the SSI_ext_ (Fig. 5h) and τ_app_ values (Fig. 5e) differ slightly between αβγ-ENaC and αβ_S520C_γ-ENaC, it cannot be ruled out that the mutation has a small effect on the gating properties of αβγ-ENaC. This also highlights one of the main drawbacks of the MTSET method: it relies on introducing a mutation whose effect on the properties of the ENaC variant under investigation cannot be known in advance. In the case of human αβγ-ENaC variants that do not differ in structural elements near the degenerin site, this effect may be negligible or at least consistent across the group of studied ENaC variants, rendering the MTSET method suitable for quantifying SSI or other gating phenomena. However, for ENaC variants from other species or ENaC variants exhibiting significantly altered gating properties, it cannot be assumed a priori that the S520C mutation has no influence on gating. For each ENaC variant, it would therefore first be necessary to determine the extent to which the S520C mutation influences gating. Given that the MTSET method is specifically intended to quantify these gating properties, this appears to be a circular argument.

Furthermore, it must be considered that the equilibrium current before MTSET-application depends not only on [Na^+^]_ex_ but can also be affected by other regulatory factors. Binding of MTSET to β-S520C could not only eliminate the inhibitory effect of [Na^+^]_ex_, but could also suppress the impact of these regulatory factors on gating. If that was the case, measuring SSI_MTSET_ would result in an overestimation of the influence of [Na^+^]_ex_ on gating.

Consequently, a method is sought that allows the determination of SSI independently of the perfusion rate and independently of any artificial modification of the ENaC subunits.

### Calculation of SSI from steady-state currents by I/V-curve fitting

An alternative strategy for investigating the influence of extracellular stimuli such as changes in [Na^+^]_ex_ on gating of ENaC variants, which in principle requires neither the introduction of a mutation nor a rapid bath exchange, is to compare Na^+^ permeabilities (P_Na_) determined from steady-state current-voltage (I/V) relationships. This strategy has already been used to investigate the ion selectivity and affinity of the allosteric Na⁺ binding site [9], as well as the influence of various mutations in the acidic cleft and the β6-β7 loop on ion selectivity of the Na⁺ binding site and the relative degree of Na⁺ self-inhibition [27].

**Fig. 6:**
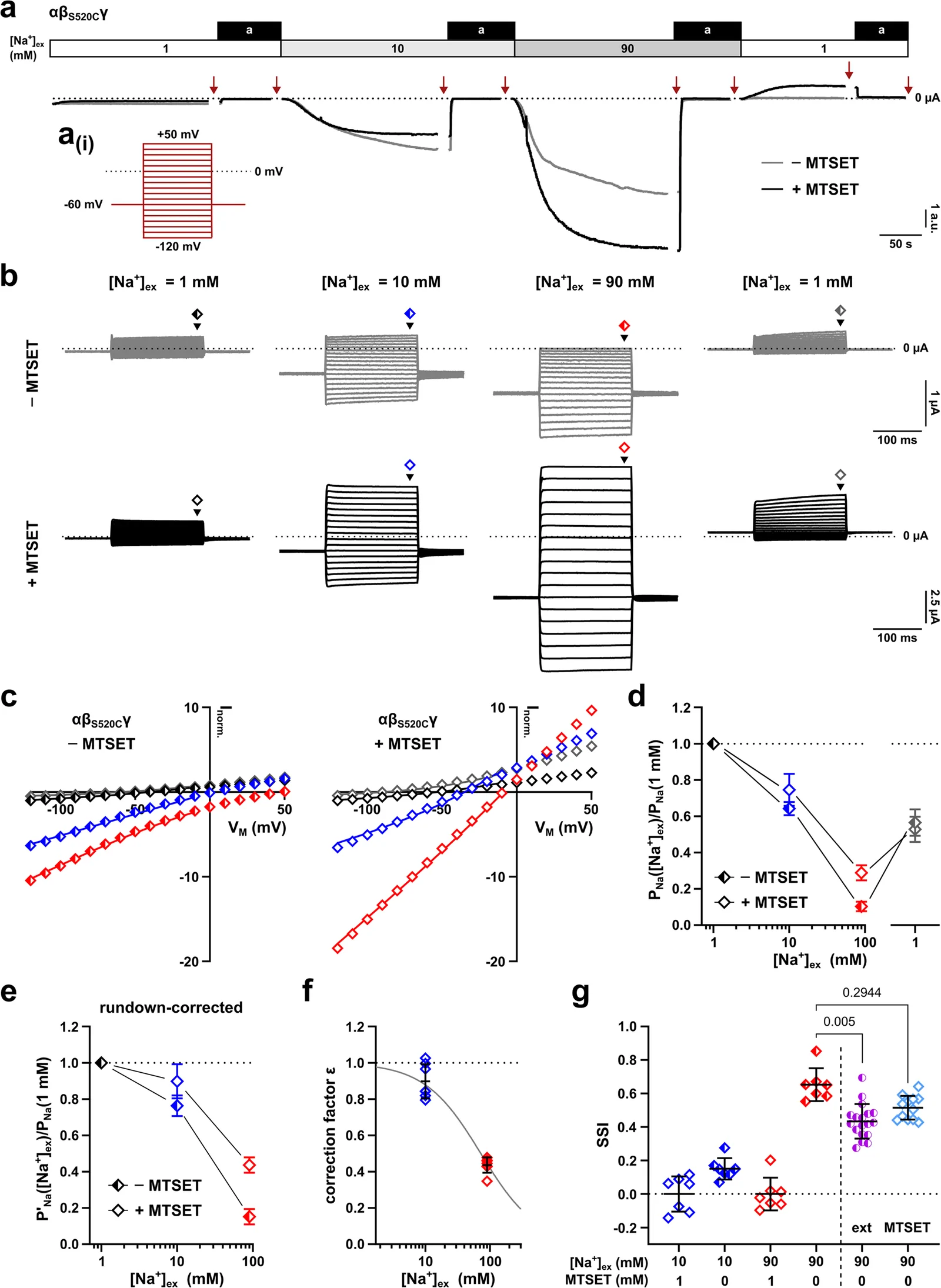
Determination of Na^+^-permeabilities and SSI of αβ_S520__C_γ-ENaC by analysing steady-state current-voltage relationships using the Goldman–Hodgkin–Katz flux model. (**a**) Representative normalised transmembrane current (I_M_) traces from automated TEVC recordings at V_M_ = −60 mV of oocytes expressing MTSET-sensitive αβ_S520C_γ-ENaC. Prior to the recording, the oocytes were incubated for 5 min in 1 mM Na^+^ solution in the absence (grey, - MTSET) or presence of 1 mM MTSET (black, + MTSET). [Na^+^]_ex_ was changed during the experiment by perfusion as indicated above the current traces. After reaching the equilibrium current, 100 µM amiloride were applied (a; black box) to fully block ENaC-mediated Na^+^-current. Red arrows indicate time points at which the continuous measurement at V_M_ = −60 mV was paused to record current responses to the voltage-step protocol shown in (**a**_**(i)**_). Dotted lines indicate the zero current and zero voltage levels. The current traces were normalised to the corresponding currents recorded at V_M_ = − 120 mV and [Na^+^]_ex_ = 1 mM. These currents were taken from the corresponding I/V relationships recorded after the first [Na^+^]_ex_ = 1 mM section and defined as one arbitrary unit (1 a.u.). (**b**) Amiloride-sensitive currents calculated from currents evoked by the voltage-step protocol in (a_(i)_) in the absence and presence of amiloride. Corresponding [Na^+^]_ex_ are indicated above the current traces. (**c**) Representative normalised steady-state current-voltage (I/V) relationships obtained from amiloride-sensitive currents in (b). All current values were normalised to the corresponding amiloride-sensitive current measured at V_M_ = −120 mV during the first [Na^+^]_ex_ = 1 mM section. Time points at which the currents were measured are highlighted in (b) with the corresponding symbols. Solid lines represent best fits with the Goldman-Hodgkin-Katz (GHK) flux equation. (**d**) [Na^+^]_ex_-dependence of relative Na^+^-permeabilities (P_Na_([Na^+^]_ex_)/P_Na_(1 mM)) of mock-treated (- MTSET, *n* = 7, *N* = 5) and MTSET-treated (+ MTSET, *n* = 7, *N* = 4) oocytes expressing αβ_S520C_γ-ENaC. P_Na_ values were obtained by fitting the GHK-flux equation to steady-state I/V relationships as shown in (c). (**e**) [Na^+^]_ex_-dependence of run-down-corrected relative Na^+^-permeabilities (P’_Na_([Na^+^]_ex_/P_Na_(1 mM)). The degree of permeability run-down was estimated for each Na^+^ concentration by assuming that P_Na_ (1 mM) decreases linearly over time. Symbols in (d) and (e) represent arithmetic mean ± SD. (**f**) [Na^+^]_ex_-dependence of the P_Na_-correction factor ε obtained from relative run-down-corrected Na^+^-permeabilities of MTSET-treated oocytes in (e). The best fit of the Michaelis-Menten Eq. 30 to the data points (grey line) yields K_M_ = 73 ± 14 mM. (**g**) SSI values of αβ_S520C_γ-ENaC determined with different methods. Left of vertical dashed line: SSI for [Na^+^]_ex_ = 10 mM and 90 mM calculated with Eq. 31 from relative Na^+^-permeabilities in (e) and correction factors in (f). Right of vertical dashed line: SSI values calculated from extrapolated peak currents of manual TEVC recordings (ext; Fig. 5a, h) and equilibrium currents before and after MTSET-treatment in automated TEVC recordings (MTSET; Fig. 5f-h). Statistical comparisons were performed using a Kruskal-Wallis test with Dunn’s multiple comparisons test. Symbols in (f) and (g) represent data points from n individual experiments; lines and error bars represent arithmetic mean ± SD

To obtain amiloride-sensitive I/V relationships at extracellular Na^+^ concentrations of 1 mM, 10 mM and 90 mM, we performed automated TEVC experiments on ENaC-expressing oocytes that had been pre-incubated in low-Na^+^ solution following the protocol shown in Fig. 6a. For each extracellular Na^+^ concentration, the voltage-step protocol shown in Fig. 6a_(i)_ was applied in the presence and absence of amiloride, which enabled the calculation of amiloride-sensitive I/V relationships. A high amiloride concentration (100 µM) was chosen to ensure full ENaC block in the entire voltage range from − 120 mV + 50 mV since the amiloride block is known to be slightly voltage-dependent [42]. Between voltage-step protocols, the transmembrane current was recorded at a holding potential of −60 mV in order to monitor the attainment of steady-state. To quantify ENaC current run-down, amiloride-sensitive I/V relationships were recorded for each oocyte in 1 mM Na^+^ solution at the beginning and the end of the protocol.

We decided to conduct the first set of experiments using the αβ_S520C_γ-ENaC mutant, as it enables us to specifically eliminate SSI by coupling MTSET to β-Cys_520_ thereby allowing us to investigate the [Na^+^]_ex_-dependence of ion permeation through the open channel pore independently of [Na^+^]_ex_-dependent gating effects. Although we did not prevent trafficking of ENaC to the membrane and thereby prevent potential replacement of MTSET-modified ENaC with unmodified ENaC delivered to the plasma membrane, the turnover of human αβ_S520C_γ-ENaC expressed in *Xenopus* oocytes is slow and amiloride-sensitive currents remain relatively stable for the employed duration of our experiments [25]. Figure 6a shows representative transmembrane currents recorded during the course of an experiment at the holding potential of V_M_ = −60 mV for both an untreated and a MTSET-pretreated oocyte expressing αβ_S520C_γ-ENaC. To better compare the effect of changes in [Na^+^]_ex_ on the current amplitude, the current traces were normalised to the steady-state current at [Na^+^]_ex_ = 1 mM. Rising [Na^+^]_ex_ from 1 mM to 10 mM and 90 mM results in an increase in the inward current in both untreated and MTSET-pretreated oocytes. As expected, at [Na^+^]_ex_ = 90 mM this increase is significantly more pronounced in MTSET-pretreated oocytes due to the MTSET-mediated elimination of self-inhibition. The same effect is also evident in the amiloride-sensitive currents (Fig. 6b) obtained using the voltage-step protocol (Fig. 6a_(i)_) and in the derived amiloride-sensitive I/V relationships (Fig. 6c). To avoid confusion, it must be noted that the apparently higher current in the untreated oocyte at [Na^+^]_ex_ = 10 mM and V_M_ = −60 mV (Fig. 6a) does not reflect a higher P_O_ of the unmodified channels, but rather the difference in the electrochemical driving force (V_M_-E_rev_): as can be seen from the normalised I/V relationships in Fig. 6c, the reversal potential of the untreated oocyte is around 0 mV at [Na^+^]_ex_ = 10 mM, whereas for the MTSET-pretreated oocyte it is around − 35 mV and thus significantly closer to V_M_ = − 60 mV.

To determine the Na^+^ permeability (P_Na_) for each condition, each I/V curve was analysed in a two-step process: First, the reversal potential (E_rev_) was determined by interpolation and then used, together with the known extracellular Na⁺ activity (a_Na,ex_), to calculate a precise value for the intracellular Na activity (a_Na,in_) with the Nernst equation (Eq. 16). In the second step, using a_Na,ex_ and a_Na,in_, P_Na_ was determined by fitting the I/V curve in the voltage range between − 120 mV and − 10 mV with the Goldman-Hodgkin-Katz (GHK) flux equation (Eq. 15). From the best fits shown in Fig. 6c, it can be seen that the GHK model is able to describe the I/V relationships within the selected voltage range accurately for all examined conditions, as has already been described by others ([9]; [14, 44]).

To investigate the effect of [Na^+^]_ex_ on P_Na_, the P_Na_ values determined for each oocyte were normalised to the corresponding P_Na_ value at [Na^+^]_ex_ = 1 mM (P_Na_(1 mM)) at the start of the protocol. Figure 6 d shows that P_Na_ decreases with increasing [Na^+^]_ex_ for both unmodified and MTSET-coupled αβ_S520C_γ-ENaC, although this decrease is, as expected, more pronounced for unmodified αβ_S520C_γ-ENaC due to SSI. However, a comparison of the P_Na_ values determined at [Na^+^]_ex_ = 1 mM at the start and end of the protocol clearly indicates that a large percentage of this decrease can be attributed to a ENaC current run-down. To estimate the extent of the current run-down for each I/V relationship, we determined from the P_Na_(1 mM) values measured at the beginning and the end of the protocol a linear time-dependent run-down factor (Eq. 17) for each oocyte, which we used to perform a time-dependent correction of the Na^+^ permeabilities. Run-down-corrected permeabilities are denoted as P’_Na_.

 Figure 6e illustrates that the normalised P’_Na_ of unmodified αβ_S520C_γ-ENaC decreases in a [Na^+^]_ex_-dependent manner from 1 at [Na^+^]_ex_ = 1 mM to 0.76 ± 0.05 at [Na^+^]_ex_ = 10 mM and finally to 0.15 ± 0.04 at [Na^+^]_ex_ = 90 mM. If this decrease was solely attributable to SSI, the P’_Na_ of MTSET-modified αβ_S520C_γ-ENaC should be [Na^+^]_ex_-independent, as the GHK model predicts that the Na⁺ permeability should only depend on the number of channels in the membrane and the single-channel open probability. Since binding of MTSET to β-Cys_520_ locks αβ_S520C_γ-ENaC in the open state (Fig. 5) and the time-dependent decrease in the number of channels has been eliminated by the run-down correction, the P’_Na_ of MTSET-modified αβ_S520C_γ-ENaC should be close to 1 for all extracellular Na⁺ concentrations. However, this is neither the case for [Na^+^]_ex_ = 10 mM nor for [Na^+^]_ex_ = 90 mM. In fact, the normalised P’_Na_ at [Na^+^]_ex_ = 10 mM and 90 mM are ~ 0.1 and ~ 0.6, respectively, lower than predicted by the GHK model (Fig. 6e). Since we had demonstrated that the coupling of MTSET to β-Cys_520_ almost completely suppresses SSI at [Na^+^]_ex_ = 90 mM (Fig. 5), the discrepancy between the measured P’_Na_ and its prediction by the GHK model must be attributed to a gating-independent [Na^+^]_ex_-dependence of the open-channel Na^+^ conductance as has been suggested by others [9, 43, 44]. It can therefore be assumed that the [Na^+^]_ex_-dependent reduction in Na^+^ permeability of the unmodified αβ_S520C_γ-ENaC is the result of a gating-dependent (SSI) and a gating-independent ([Na^+^]_ex_-dependent single-channel permeability) component. P’_Na_ is then given for any [Na^+^]_ex_ by:

28 $$\begin{array}{c}P'_{Na}\left({\left[Na^+\right]}_{ex}\right)\\=P_{Na}^0\cdot\varepsilon\left({\left[Na^+\right]}_{ex}\right)\cdot\left(1-SSI\left({\left[Na^+\right]}_{ex}\right)\right)\end{array}$$

with P^0^_Na_ and ε([Na^+^]_ex_) being the Na^+^ permeability at [Na^+^]_ex_ = 0 mM and the [Na^+^]_ex_-dependent correction factor, respectively. ε([Na^+^]_ex_) accounts for the [Na^+^]_ex_-dependence of the open-channel Na^+^ permeability. With P^0^_Na_ ≈ P_Na_(1 mM) we can calculate the [Na^+^]_ex_-dependent correction factor from the P’_Na_ values of MTSET-modified αβ_S520C_γ-ENaC as follows:

29 $$\:\varepsilon\left({\left[{Na}^+\right]}_{ex}\right)={\left(\frac{{P'}_{Na}\left({\left[{Na}^+\right]}_{ex}\right)}{P_{Na}\left(1\:mM\right)}\right)}_{MTSET}$$

This gives ε = 0.90 ± 0.09 for [Na^+^]_ex_ = 10 mM and ε = 0.44 ± 0.04 for [Na^+^]_ex_ = 90 mM (Fig. 6f). As previously suggested by Palmer and Frindt [44], the reduction of P_Na_ described by ε can be explained by the saturation of a Na^+^ binding site within the ion permeation pathway. Thus, an increase in [Na^+^]_ex_ has an apparent inhibitory effect on P_Na_, which is why ε can be expressed as:

30 $$\varepsilon=1-\frac{{\left[Na^+\right]}_{ex}}{{\left[Na^+\right]}_{ex}+K_M}$$

where K_M_ is the apparent Michaelis-Menten constant (A simplified explanation for the Michaelis-Menten-like saturating behaviour is given in Appendix S2). This model predicts (outlined in Appendix S3) that the single-channel amplitude should approach a constant maximal value as [Na^+^]_ex_ increases. Such saturation has indeed been observed for ENaC in several studies [41, 43, 44]. Fitting Eq. 30 to the experimentally determined ε values in Fig. 6f yields an apparent K_M_ value of 73 ± 14 mM.

Assuming that the [Na^+^]_ex_-dependence of the open-channel Na^+^ permeability is identical for unmodified and MTSET-coupled αβ_S520C_γ-ENaC, SSI can be estimated from P’_Na_ as follows:

31 $$SSI_{IV}({\left[Na^+\right]}_{ex})=1-\frac1\varepsilon\cdot\frac{P'_{Na}\left({\left[Na^+\right]}_{ex}\right)}{P_{Na}(1\;mM)}$$

The SSI_IV_ values, calculated for unmodified αβ_S520C_γ-ENaC using the mean ε values shown in Fig. 6f, are 0.15 ± 0.06 and 0.65 ± 0.10 (*n* = 7) at [Na^+^]_ex_ = 10 mM and 90 mM, respectively (Fig. 6 g). Thus, the SSI_IV_ value determined from P’_Na_ at [Na^+^]_ex_ = 90 mM is slightly higher than the SSI values determined for the αβ_S520C_γ-ENaC mutant using the extrapolation method and the MTSET method (Fig. 6 g). It should be noted that the standard deviations σ_SSI_ of the SSI_IV_ values calculated in this way are influenced exclusively by the scattering of the individual run-down-corrected relative permeabilities P’_Na_([Na^+^]_ex_)/P_Na_(1 mM), while the uncertainty in the ε values is not taken into account. To estimate the impact of the uncertainty in ε on σ_SSI_ at [Na^+^]_ex_ = 10 mM and 90 mM, we have additionally calculated the standard deviations of SSI_IV_ by error propagation, as described in Appendix S5. This analysis yielded a σ_SSI_ value of 0.10 for the SSI_IV_ values at both Na^+^ concentrations, suggesting that the uncertainty in ε has only a minor impact on the uncertainty of the calculated SSI_IV_ values.

**Fig. 7:**
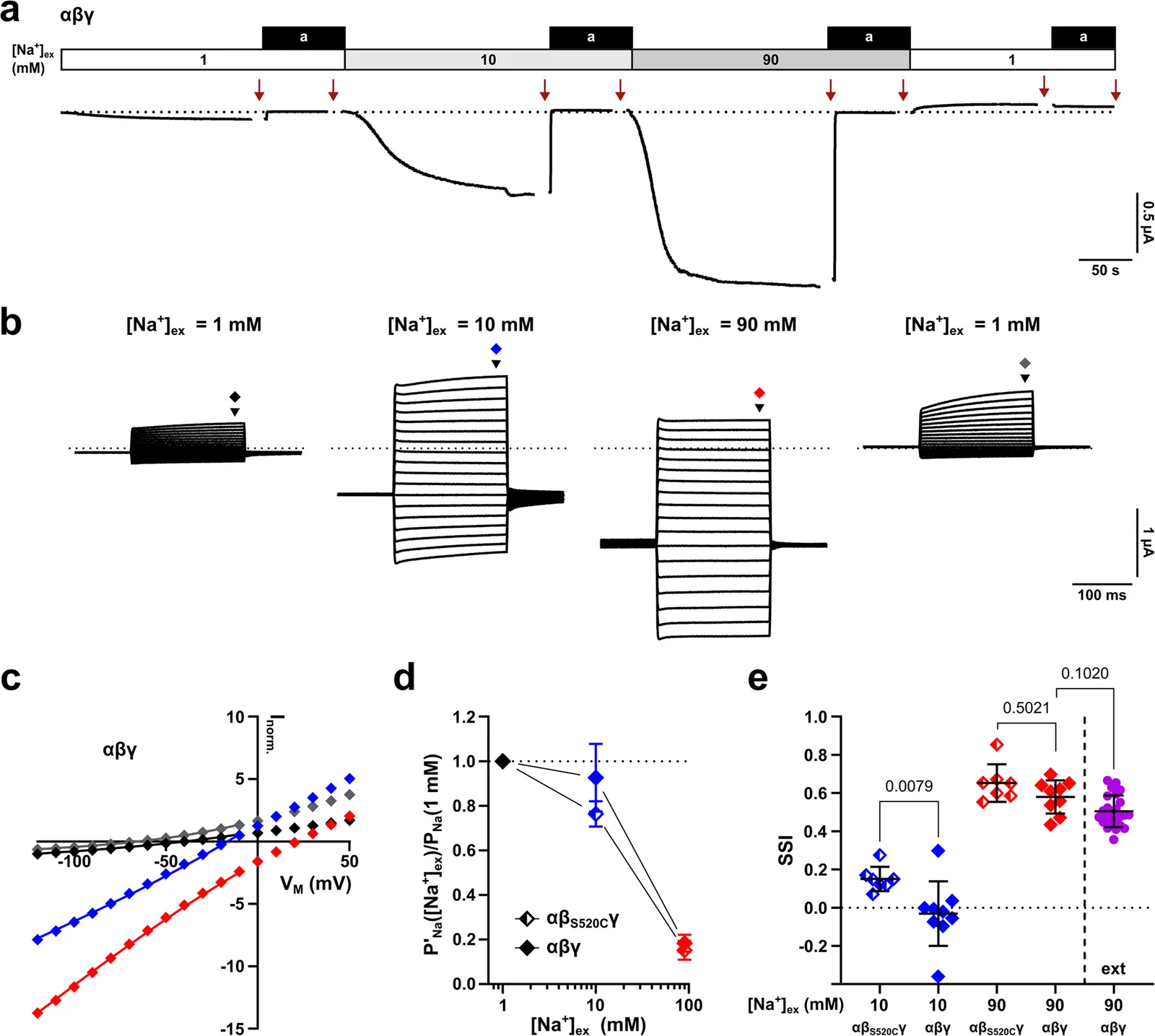
Determination of Na^+^-permeabilities and SSI of αβγ-ENaC by analysing steady-state current-voltage relationships using the Goldman–Hodgkin–Katz flux model.(**a**) Representative transmembrane current (I_M_) trace from an automated TEVC recording at V_M_ = −60 mV of an oocyte expressing αβγ-ENaC. [Na^+^]_ex_ was changed during the experiment by perfusion as indicated above the current trace. After reaching the equilibrium current, 100 µM amiloride were applied (a; black box) to fully block ENaC-mediated Na^+^-current. Red arrows indicate time points at which the continuous measurement at V_M_ = −60 mV was paused to record current responses to the voltage-step protocol shown in Fig. 6a_(i)_. (**b**) Amiloride-sensitive currents calculated from currents evoked by the voltage-step protocol in Fig. 6a_(i)_ in the absence and presence of amiloride. Corresponding [Na^+^]_ex_ are indicated above the current traces. Dotted lines in (a) and (b) indicate zero current levels. **(c)** Representative normalised steady-state current/voltage (I/V) relationships obtained from amiloride-sensitive currents in (b). All current values were normalised to the amiloride-sensitive current measured at V_M_ = −120 mV during the first [Na^+^]_ex_ = 1 mM section. Time points at which the currents were measured are highlighted in (b) with the corresponding symbols. Solid lines represent best fits with the Goldman-Hodgkin-Katz (GHK) flux equation. (**d**) [Na^+^]_ex_-dependence of run-down-corrected relative Na^+^-permeabilities (P’_Na_([Na^+^]_ex_)/P_Na_(1 mM)) of oocytes expressing αβ_S520C_γ-ENaC (*n* = 7, *N* = 5) and αβγ-ENaC (*n* = 9, *N* = 4). P_Na_ values were obtained by fitting the GHK-flux equation to steady-state I/V relationships as shown in (c). Symbols represent arithmetic mean ± SD. **(e)** SSI values of αβ_S520C_γ-ENaC and αβγ-ENaC determined with different methods. Left of vertical dashed line: SSI for [Na^+^]_ex_ = 10 mM and 90 mM calculated with Eq. 31 from relative run-down-corrected Na^+^-permeabilities obtained by I/V-curve fitting as in (c) and correction factors in Fig. 6f. Right of vertical dashed line: SSI value of αβγ-ENaC calculated from extrapolated peak currents of manual TEVC recordings (ext; Fig. 3a, c). Statistical comparison between SSI values for [Na^+^]_ex_ = 10 mM was performed using a Mann-Whitney test. Statistical comparisons between SSI values for [Na^+^]_ex_ = 90 mM were performed using a Kruskal-Wallis test with Dunn’s multiple comparisons test. Symbols represent data points from n individual experiments; lines and error bars represent arithmetic mean ± SD

Next, using the same experimental procedure as shown in Fig. 6a, we recorded amiloride-sensitive steady-state I/V curves from oocytes expressing wild-type αβγ-ENaC as described above. Transmembrane currents recorded during a representative experiment at the holding potential of V_M_ = −60 mV and in response to the voltage-step protocol (Fig. 6a_(i)_) are shown in Fig. 7a and b, respectively. As expected, rising [Na^+^]_ex_ from 1 mM to 10 mM and 90 mM results in a gradual increase in the inward current and to a shift of E_rev_ to more positive potentials as can been seen from the amiloride-sensitive I/V relationships (Fig. 7c). The I/V curves recorded from *n* = 9 oocytes expressing wild-type αβγ-ENaC were fitted in the voltage range between − 120 mV and − 10 mV with the GHK flux equation (Eq. 15) to obtain the run-down-corrected Na^+^-permeabilities P’_Na_, as described above. Figure 7d illustrates that the P’_Na_ values of wild-type αβγ-ENaC exhibit a similar [Na^+^]_ex_ dependence as the P’_Na_ values of the unmodified αβ_S520C_γ-ENaC mutant. At [Na^+^]_ex_ = 90 mM, the normalised P’_Na_ values are 0.18 ± 0.04 and 0.15 ± 0.04 for the wild-type and the mutant channel, respectively, and are therefore essentially identical. Using the mean correction factor ε determined above, Eq. 31 yields an SSI_IV_ value of 0.58 ± 0.09 at [Na^+^]_ex_ = 90 mM for wild-type αβγ-ENaC, which differs only slightly and not significantly from the SSI_IV_ value obtained for the αβ_S520C_γ-ENaC mutant (SSI_IV_ = 0.65 ± 0.10) (Fig. 7e). However, analysis of the I/V curves recorded at [Na^+^]_ex_ = 10 mM suggests that the wild-type channel, unlike the mutant, is not inhibited by 10 mM extracellular Na^+^ (SSI_IV_ = −0.03 ± 0.17). This discrepancy between αβγ- and αβ_S520C_γ-ENaC might be attributed to either a true effect of the mutation on SSI at low extracellular Na^+^ concentrations or an inaccuracy in the estimation of the current run-down.

Notably, SSI_IV_ and SSI_ext_ obtained for the wild-type channel at [Na^+^]_ex_ = 90 mM are consistent (SSI_IV_ = 0.58 ± 0.09 and SSI_ext_ = 0.50 ± 0.08, Fig. 7e), confirming that the method developed here for determining SSI from steady-state I/V relationships is suitable as an alternative to the analysis of transient currents from [Na^+^]_ex_-jump experiments.

## Discussion

In this study, we evaluated and developed experimental and analytical methods for the reliable quantification of sodium self-inhibition (SSI) of the epithelial sodium channel (ENaC) using manual and automated TEVC systems. The establishment of automated electrophysiological protocols to accurately quantify SSI is essential for both the investigation of ENaC variants with unknown disease significance and the biophysical characterisation of rationally designed mutants aiming to explore ENaC gating mechanisms.

Although the patch-clamp technique can undoubtedly be considered the gold standard for the electrophysiological characterisation of ion channels, manual TEVC on *X. laevis* oocytes is a widely used method for studying ENaC and many other ion channels and transporters. We have previously demonstrated that automated TEVC systems can be generally used to record ENaC SSI [33], however, to the best of our knowledge, there are no established protocols that enable precise quantification of SSI on a medium- to high-throughput scale, taking into account specific limitations of automated TEVC systems. In most publications, ENaC SSI is calculated as apparent SSI (SSI_app_) derived from the transient transmembrane current recorded in response to a rapid increase in the extracellular Na^+^ concentration ([Na^+^]_ex_-jump). This method assumes that the solution exchange occurs significantly faster than the allosteric inhibition of ENaC by extracellular Na^+^. The peak current, which occurs shortly after the [Na^+^]_ex_-jump, can therefore be interpreted as the current passing through the still fully active channels with the [Na^+^]-gradient being completely established. We conducted such [Na⁺]_ex_-jump experiments using both a manual and a 96-well plate-based automated TEVC system on oocytes expressing human αβγ-ENaC. SSI_app_ values determined using the automated system were systematically lower than those obtained with the manual system. Using numerical simulations, we demonstrate that this discrepancy can be attributed to the lower perfusion rate in the automated system. In our view, this discrepancy cannot simply be resolved by increasing the nominal perfusion rate since the local solution exchange rate near the oocyte also depends on the oocyte itself (e.g., the vitelline layer providing a potentially unstirred environment near the membrane) and the geometry of the recording chamber. While manual systems typically use elongated recording chambers in which convection-dominated fluid flow enables rapid solution exchange around the oocyte, fluid exchange in well-based recording chambers occurs primarily through mixing and diffusion, leading to gradual rather than stepwise buffer changes. Furthermore, our numerical simulations revealed that, even at high perfusion rates achievable in manual TEVC systems, SSI_app_ can deviate significantly from true SSI (SSI_true_). Consequently, SSI_app_ cannot be considered a reliable estimate of SSI_true_, neither in automated nor in manual TEVC recordings.

We therefore investigated whether the true peak current, and thus SSI_true,_ can be estimated by backward extrapolation of the exponential current decay of transient currents elicited by the [Na^+^]_ex_-jump. This approach was proposed by Chraïbi and Horisberger [14], but has only been used to determine SSI in a few studies [9, 23], likely because this extrapolation method was never systematically evaluated. We applied the extrapolation method to numerically simulated [Na^+^]_ex_-jump experiments in which we systematically varied the kinetics of Na^+^ inhibition and the perfusion rate. The SSI value determined using the extrapolation method (SSI_ext_) was always closer to SSI_true_ than the corresponding SSI_app_. The deviation of SSI_ext_ from SSI_true_ was generally less than 0.1, as long as the kinetics of the solution exchange were faster than the kinetics of the allosteric Na^+^ inhibition (τ_exchange_ > τ_true_). This is usually the case if the current response to a [Na^+^]_ex_-jump exhibits a pronounced peak. Based on these results, we strongly recommend applying the extrapolation method to transient current recordings to improve the reliability of the obtained SSI values.

Importantly, the extrapolation method can only yield reliable results if the exponential phase of the current decay is not significantly distorted. This is the case whenever the kinetics of the Na^+^ inhibition are similarly fast or even faster than the solution exchange. For ENaC variants with very rapid Na⁺ inhibition kinetics, the peak might even disappear entirely, falsely implying absence of SSI. This highlights that the extrapolation method does not resolve the general problem of the dependence of transient current recordings on the perfusion rate. We therefore explored two methods that enable the calculation of SSI from steady-state currents: (1) the selective elimination of Na^+^ inhibition by chemical modification, and (2) the calculation of SSI from steady-state Na⁺ permeabilities.

The elimination of Na^+^ inhibition was achieved by covalent coupling of the sulfhydryl reagent MTSET to a cysteine introduced into the ENaC β-subunit (S520C). The steady-state currents before and after MTSET coupling allowed to calculate an SSI value (SSI_MTSET_) that was almost identical to the SSI_ext_ value we had determined for wild-type αβγ-ENaC by applying the extrapolation method to [Na^+^]_ex_-jump experiments performed with the manual TEVC system. The MTSET method may therefore be a practical alternative to transient current recordings in automated TEVC systems with limited perfusion rates. However, this method requires the introduction of the S520C mutation into the β-subunit. For human αβγ-ENaC examined here, the effect of this mutation on the electrophysiological properties of the channel was negligible; however, this may not apply to every ENaC variant. Furthermore, it cannot necessarily be assumed that coupling of MTSET to the β-subunit has the same effect in all ENaC variants, i.e. that it completely reverses Na^+^ inhibition without affecting the single-channel conductance for Na^+^ or other regulatory mechanisms. Another factor complicating the use of MTSET is its instability in aqueous buffers: MTSET hydrolyses at neutral pH and room temperature with a half-life of around 10 min [26]. Hence, pH-buffered solutions containing MTSET must be prepared immediately before use and cannot be stored for long time periods in the reservoir of an automated TEVC system.

The second and, in our view, most promising approach for investigating ENaC SSI in automated systems is the analysis of amiloride-sensitive steady-state I/V relationships recorded at various extracellular Na^+^ concentrations. Similar approaches have already been used to investigate the properties of the allosteric Na^+^ binding site in the ENaC α-subunit ([9]; [27]). However, to the best of our knowledge, such an approach has never been used to quantitatively determine SSI.

The key challenge with this approach is that the extracellular Na^+^ concentration influences the I/V relationship of the ENaC-mediated Na^+^ current in two ways: (1) by altering the electrochemical driving force for the passive Na^+^ flux through the open ENaC pore; and (2) by altering the probability of finding a channel in the open state.

Therefore, to quantify the influence of [Na⁺]_ex_ on gating, a mathematical model that adequately describes the Na^+^ flux through the open ENaC pore as a function of the membrane potential and the Na⁺ concentrations on both sides of the membrane is required. Since there is currently no kinetic permeation model for ENaC, we used the GHK constant-field model. Fitting the GHK flux equation to the experimentally obtained steady-state I/V relationships yields a permeability coefficient P_Na_ which describes how easily Na^+^ can pass through the membrane. Based on the GHK model’s prediction that P_Na_ depends solely on the single-channel open probability and the number of channels in the membrane, we reasoned that the inhibitory effect of [Na^+^]_ex_ on gating (i.e. SSI) can be directly calculated from the P_Na_ values determined under different [Na^+^]_ex_ conditions. In a series of experiments using the MTSET-sensitive αβ_S520C_γ-ENaC mutant, which enabled us to specifically eliminate SSI, we were able to show that the influence of [Na^+^]_ex_ on gating can indeed be measured as a reduction in P_Na_. However, we found that two additional [Na^+^]_ex_-dependent factors must be taken into account: (1) the time-dependent current run-down; and (2) the [Na^+^]_ex_-dependent reduction in open single-channel permeability.

The current run-down, which we have considered by introducing the correction factor f_corr_, is very likely the result of [Na^+^]_in_-dependent feedback inhibition, which leads to ENaC retrieval from the plasma membrane [48, 59]. The link between run-down and [Na^+^]_in_ is supported by the finding that the extent of the current run-down at the end of the experiment correlates positively with the activity of accumulated intracellular Na^+^ (Supplementary Figure S5c). This Na^+^ accumulation, in turn, depends on the ENaC-mediated Na^+^ influx during the experiment and, consequently, also on [Na^+^]_ex_. This connection is illustrated by the comparison of intracellular Na⁺ activities of MTSET-pretreated and untreated αβ_S520C_γ-ENaC-expressing oocytes, obtained by analysing reversal potentials of steady-state I/V relationships: in the case of untreated oocytes, the intracellular Na⁺ activity rose from approximately 5 mM at the beginning of the experiments to 21.6 ± 10.4 mM at [Na^+^]_ex_ = 90 mM. In contrast, the intracellular Na⁺ activity in MTSET-treated oocytes increased to 57.1 ± 25.5 mM at [Na^+^]_ex_ = 90 mM (Supplementary Figure S5a). The significantly greater Na⁺ accumulation in MTSET-pretreated oocytes is most certainly the result of a larger Na⁺ influx caused by the MTSET-induced elimination of SSI.

Since the temporal course of the run-down can only be estimated and is therefore inevitably error-prone, intracellular Na^+^ accumulation should be reduced to a minimum by limiting the Na^+^ influx. This can be achieved by (1) minimising the duration of the experiment; (2) reducing the ion channel density in the plasma membrane through the injection of lower amounts of cRNA; and (3) by setting the holding potential applied between the voltage-step protocols to a less negative value to reduce the driving force for Na^+^ influx. As the current run-down can probably only be minimised, but not eliminated, the magnitude of the error with which the run-down-corrected permeabilities can be determined depends heavily on the used run-down model. The linear interpolation employed here certainly represents a simplification and should, if possible, be replaced by an empirically validated model.

To account for the [Na^+^]_ex_-dependent reduction of the open single-channel permeability, we introduced the correction factor ε. Unlike f_corr_, the influence of ε on P_Na_ cannot be reduced by altering the experimental conditions, as ε is a consequence of the mechanism of ion permeation through the open ENaC pore. ε reflects the Michaelis-Menten-like saturation of the single-channel conductance, which has previously been observed in single-channel recordings [41, 43, 44]. Remarkably, in a series of patch-clamp experiments in which the extracellular NaCl was also replaced isosmotically with NMDG-Cl, Palmer and Frindt [43] were able to determine a K_M_ value of 75 mM extracellular Na^+^ for the saturation of the single-channel conductance of amiloride-sensitive Na⁺ channels in the apical membrane of the rat cortical collecting tubule. This value is remarkably similar to the K_M_ value of 73 ± 14 mM found here.

Mechanistically, the Michaelis-Menten-like [Na^+^]_ex_-dependence of ε can be interpreted as the saturation of a single Na^+^ binding site within the ion permeation pathway, or as the saturation of the occupancy of a specific state in the ion transport cycle. Such saturation occurs when ion permeation is sorption-limited, i.e. the association and/or dissociation of an ion at or from the pore represents the rate-limiting step, while ion translocation within the pore occurs relatively rapidly [38]. The GHK model is unable to predict sorption-limited permeation, as it assumes that ions at the membrane surfaces are in instantaneous equilibrium with the aqueous solutions. Association and dissociation are therefore not explicitly modelled. By introducing ε, we were able to overcome this limitation. However, it must be emphasised that this correction is based solely on empirical evidence and ignores the influence of both the membrane potential and the intracellular Na^+^ concentration. Future efforts should therefore aim to develop a permeation model (e.g., in the form of a kinetic Markov state model) that links experimentally observable properties of ion flux through the ENaC pore to structural information, as it has already been demonstrated for K^+^ channels [8, 51, 62].

One limitation of our method is that the [Na^+^]_ex_-dependence of the correction factor ε was determined using the αβ_S520C_γ-ENaC mutant, as it cannot be ruled out that both the β-S520C mutation and the coupling of MTSET to the introduced cysteine residue influence the [Na^+^]_ex_-dependence of the single open-channel permeability. To eliminate this uncertainty, the [Na^+^]_ex_-dependence of ε could be determined by analysing single-channel recordings of the wild-type αβγ-ENaC. These data could then be used to develop a detailed permeation model. However, a remaining limitation is that this method can only be used to determine the effect of a change in [Na^+^]_ex_ on gating if the mutation of interest does not alter the mechanism of ion permeation. As soon as this assumption can no longer be justified (e.g. because the mutation of interest lies within the permeation pathway), only the calculated permeabilities can be compared.

In summary, we have shown that determining SSI of ENaC variants using microplate-based automated TEVC systems is complicated by limitations in solution exchange. We therefore investigated methods that enable the determination of SSI despite these limitations: (1) extrapolation of the true peak current in transient current recordings; (2) comparison of steady-state currents before and after elimination of the SSI via MTSET coupling; and (3) analysis of steady-state I/V relationships recorded at different extracellular Na⁺ concentrations. Using the latter two methods, mean SSI values between 0.5 and 0.6 were determined for human αβγ-ENaC, whereas the extrapolation method yielded a significantly lower value of approximately 0.3. Although the use of the extrapolation method reduces the influence of the perfusion rate on the determined SSI value, it cannot eliminate it entirely. Therefore, to determine the SSI of ENaC variants using perfusion rate-limited automated TEVC systems, a method based on the analysis of steady-state currents should be employed. The analysis of I/V relationships is preferable to the use of MTSET, as it does not require the introduction of additional mutations and consequently relies on fewer assumptions. Furthermore, ENaC P_O_ in *Xenopus* oocytes can be affected by mechanical forces, including shear-forces triggered by perfusion during TEVC experiments [4, 31]. The analysis of I/V relationships from steady state currents obtained after pausing perfusion allows for quantification of Na^+^ permeabilities and SSI without the influence of shear-force on ENaC P_O_. This may prevent further discrepancies in SSI estimated from manual and automated TEVC recordings.

## Supplementary Information

Below is the link to the electronic supplementary material.

Supplementary Material 1 (PDF 10.1 MB)

## Data Availability

Data are made available in the manuscript, and as Source Data deposited at the Zenodo data depository (10.5281/zenodo.21626248).
