# Supplementary material for: Analysis of sodium self-inhibition of the epithelial sodium channel (ENaC) using classical and medium-throughput automated two-electrode voltage-clamp recordings
Source: Pflugers Arch. 2026 Sep 28;478(10):82. doi: 10.1007/s00424-026-03212-w (PMC13616839; doi:10.1007/s00424-026-03212-w)
Supplement: Supplementary file 1 [file 424_2026_3212_MOESM1_ESM.pdf]

Supplementary Information

Analysis of sodium self-inhibition of the epithelial sodium channel (ENaC) using classical and medium-throughput automated two-electrode voltage-clamp recordings

Karen Lizet Luján López[1,2], Nadja Furtwängler[1], Carol Merlin Frank[1], Mike Althaus[1#] and Oliver Rauh[1$]

1 Institute for Functional Gene Analytics, Bonn-Rhein-Sieg University of Applied Sciences, Rheinbach, Germany

2 Institute for Animal Physiology, Justus-Liebig-University, Giessen, Germany

$ Correspondence:

Dr. Oliver Rauh

Institute for Functional Gene Analytics

Bonn-Rhein-Sieg University of Applied Sciences Von-Liebig-Str. 20

53359 Rheinbach, Germany

E-Mail: oliver.rauh@h-brs.de

# Correspondence:

Prof. Dr. Mike Althaus

Institute for Functional Gene Analytics

Bonn-Rhein-Sieg University of Applied Sciences Von-Liebig-Str. 20

53359 Rheinbach, Germany

E-Mail: mike.althaus@h-brs.de

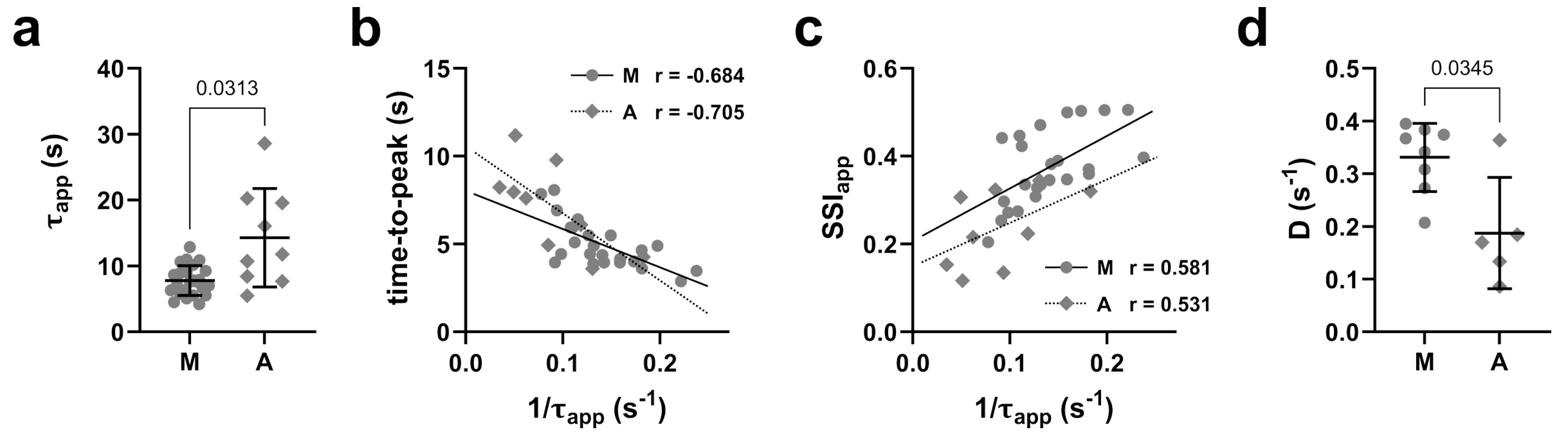

Supplementary Figure S1: Comparative analysis of transient currents recorded in response to a $[Na^+]_{ex}$-jump from 1 to 90 mM using manual and automated TEVC. (a) Apparent time constants (τ_{app}) of the post-peak current decay from manual (M; n = 24, N = 6) and automated (A; n = 9, N = 3) TEVC recordings shown in **Figure 1.** τ_{app} values were obtained by fitting of a single exponential function (**Equation 11**) to the current between the inflection point of the post-peak current and the time at which about 80% of the current decay had occurred. **(b, c)** Scatter plots of $1/\tau_{app}$ versus corresponding time-to-peak **(b)** or corresponding SSI_{app} **(c)** from manual and automated TEVC recordings shown in main **Figure 1.** Solid and dotted lines represent best fits of a linear function to data from manual (M) and automated (A) TEVC recordings, respectively. Corresponding Pearson correlation coefficients (r) are provided in the graphs. **(d)** Perfusion rates D of the manual (M; n = 8, N = 3) and automated (A; n = 5, N = 1) TEVC systems used for the experiments of this study determined by analysing the rising phase of ENaC-mediated transient currents under conditions in which Na^+ self-inhibition has been abolished either by pretreatment with MTSET or chymotrypsin (for details see **Appendix S4**). D values were obtained by fitting **Equation A4.3** to the current of the rising phase as shown in **Supplementary Figure S6**. Statistical analyses in (a) and (d) were performed using unpaired Student's t-test with Welch's correction.

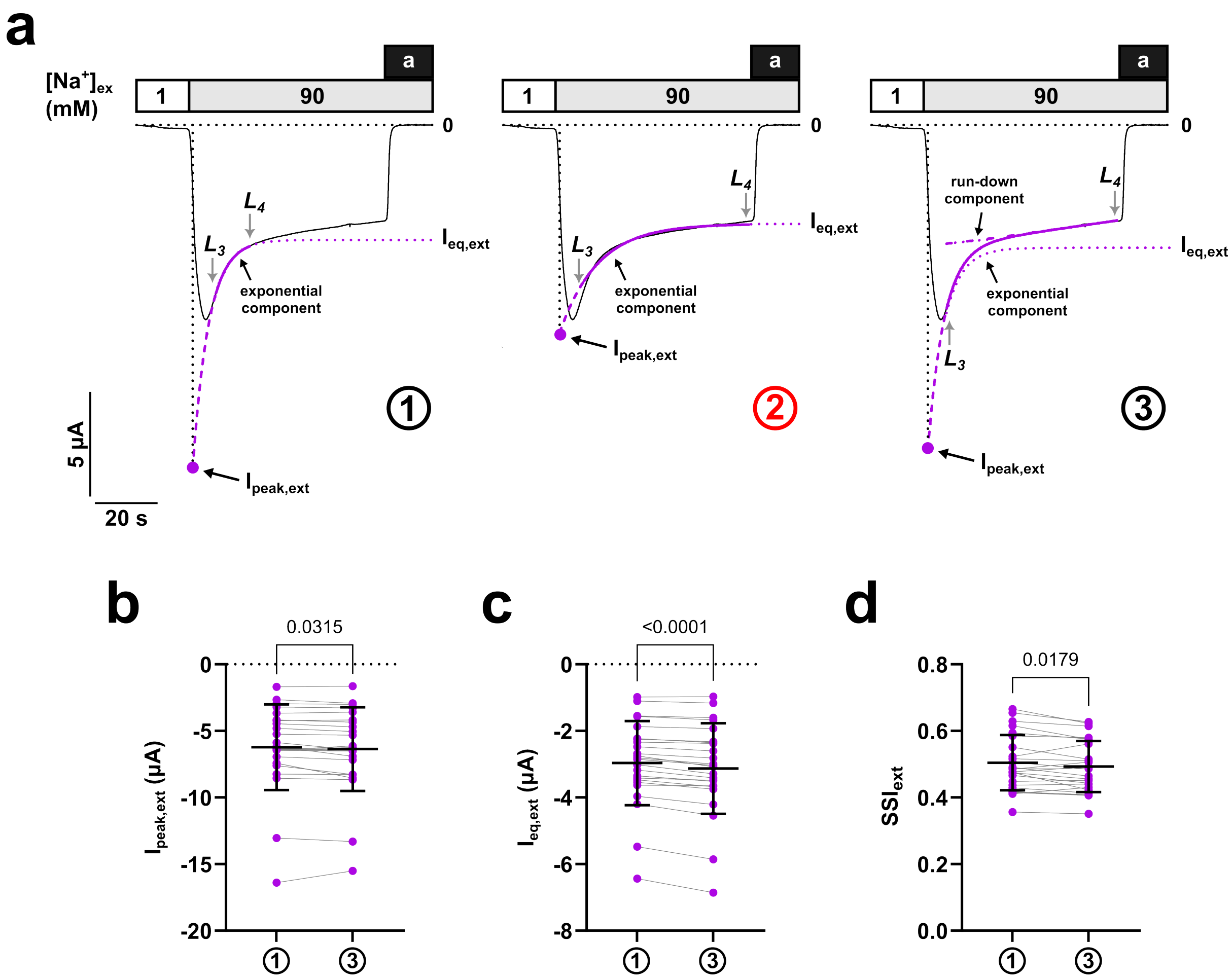

Supplementary Figure S2: Impact of current run-down on the results of the peak-extrapolation method. (a) Representative current response of an oocyte expressing αβγ-ENaC to a $[Na^+]_{ex}$-jump from 1 mM to 90 mM recorded using manual TEVC. The inflection point of the rising phase was determined as shown in **Figure 3a** and set to t = 0. To estimate the true peak current by extrapolation, the post-peak current was analysed in three ways: (1) Fitting of a single exponential function (solid purple line, **Equation 11**) to the current between the inflection point of the falling phase (L_3) and the time at which about 80% of the current decay had occurred (L_4); (2) Fitting of a single exponential function (solid purple line, **Equation 11**) to the current between the inflection point of the falling phase (L_3) and the current about 60 s after the $[Na^+]_{ex}$-jump (L_4); (3) Fitting of **Equation 12**, which combines the exponential current decay with a time-dependent linear channel run-down, to the current between the inflection point of the falling phase (L_3) and the current about 60 s after the $[Na^+]_{ex}$-jump (L_4). For approach (3), the exponential and run-down components of the best fit are shown separately. Approaches (1) and (3) provide good fits within the respective interval [L_3, L_4], while approach (2) fails. **(b-d)** Comparison of parameters $I_{peak,ext}$ **(b)**, $I_{eq,ext}$ **(c)**, and SSI_{ext} **(d)** obtained by analysing n = 24 (N = 6) transient current recordings using approaches (1) and (3). Data points connected by grey lines belong to the same current trace; black lines and error bars represent arithmetic mean ± SD. Statistical comparisons were performed using a Wilcoxon matched-pairs signed rank test (b, c) or a Student's paired t-test (d).

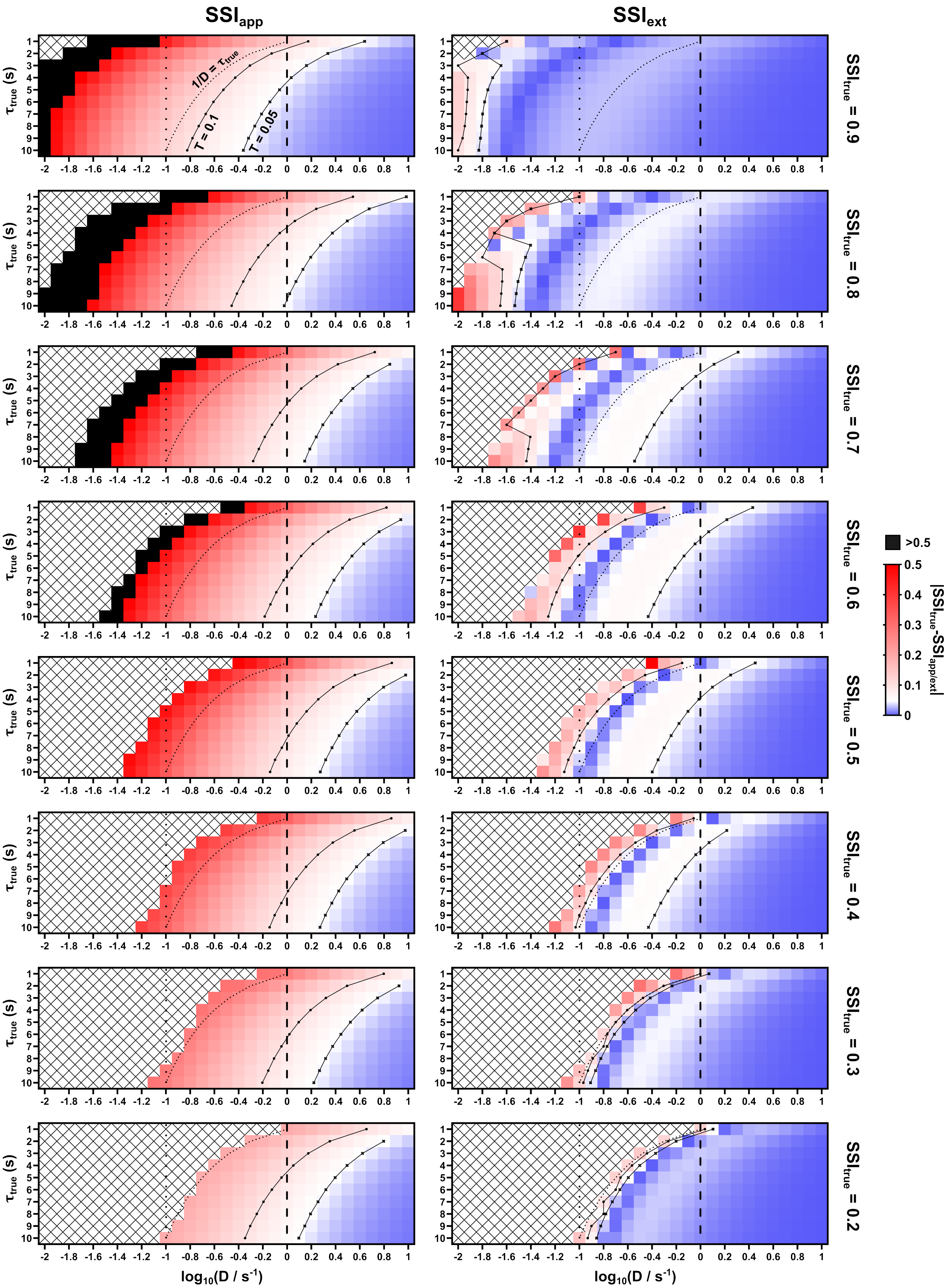

Supplementary Figure S3: Examination of the impact of Na^+ inhibition kinetics (τ_{true}) and perfusion rate (D) on the deviation of SSI_{app} and SSI_{ext} from SSI_{true} using numerical simulations. The heatmaps show the absolute value of the deviation of SSI_{app} **(left)** and SSI_{ext} **(right)** from SSI_{true} ($|SSI_{true}-SSI_{app}|$ and $|SSI_{true}-SSI_{ext}|$, respectively) depending on the perfusion rate D and the true time constant of current decay τ_{true}. The two heatmaps shown side by side in a row come from the same set of simulations, which were run using the SSI_{true} value specified on the right. Each row of a heatmap corresponds to a curve as in main **Figure 4b**. Since D

was varied exponentially for numerical simulations, the x-axis shows the common logarithm of D. The range of perfusion rates typically used in TEVC recordings is indicated by a vertical dotted line (lower limit: 0.1 s^{-1}) and a vertical dashed line (upper limit: 1 s^{-1}). Perfusion rates $D_{T=0.05}$ and $D_{T=0.1}$ at which the deviation $|SSI_{true}-SSI_{app}|$ or $|SSI_{true}-SSI_{ext}|$ for a given τ_{true} value first exceeds the threshold T = 0.05 and T = 0.1 are plotted as connected black crosses and connected black circles, respectively. The curved dotted line marks those D values for which the time constant of the solution exchange ($\tau_{exchange}$ = 1/D) is identical to τ_{true}. Colour code: $|SSI_{true}-SSI_{app/ext}| < 0.05$, shades of blue; $|SSI_{true}-SSI_{app/ext}| = 0.05$, white; $|SSI_{true}-SSI_{app/ext}| > 0.05$, shades of red; $|SSI_{true}-SSI_{app/ext}| > 0.5$, black. Black crosses indicate τ_{true}/D combinations that did not result in a peak in the simulated current trace.

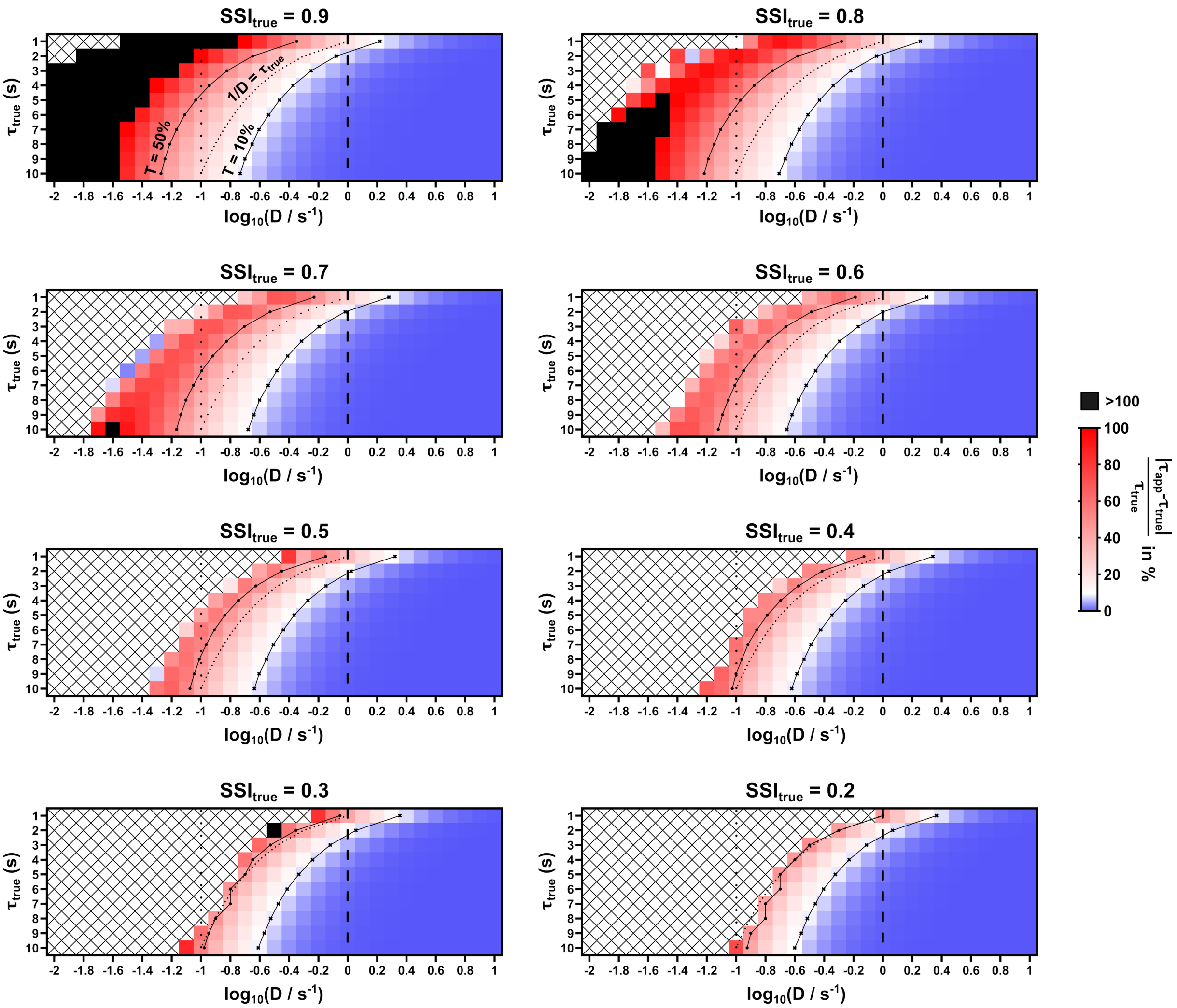

Supplementary Figure S4: Examination of the impact of Na^+ inhibition kinetics (τ_{true}) and perfusion rate (D) on the deviation of τ_{app} from τ_{true} using numerical simulations. The heatmaps show the deviation of τ_{app} from τ_{true} ($|\tau_{app}-\tau_{true}|/\tau_{true}$) for SSI$_{true}$ values specified above each graph depending on the perfusion rate D and the true time constant of current decay τ_{true}. Since D was varied exponentially for numerical simulations, the x-axis shows the common logarithm of D. The range of perfusion rates typically used in TEVC recordings is indicated by a vertical dotted line (lower limit: 0.1 s^{-1}) and a vertical dashed line (upper limit: 1 s^{-1}). Perfusion rates $D_{T=10\%}$ and $D_{T=50\%}$ at which the deviation $|\tau_{app}-\tau_{true}|/\tau_{true}$ for a given τ_{true} value first exceeds the threshold T = 10% and T = 50% are plotted as connected black crosses and connected black circles, respectively. The curved dotted line marks those D values for which the time constant of the solution exchange ($\tau_{exchange}$ = 1/D) is identical to τ_{true}. Colour code: $|\tau_{app}-\tau_{true}|/\tau_{true}$ < 10%, shades of blue; $|\tau_{app}-\tau_{true}|/\tau_{true}$ = 10%, white; $|\tau_{app}-\tau_{true}|/\tau_{true}$ > 10%, shades of red; $|\tau_{app}-\tau_{true}|/\tau_{true}$ > 100%, black. Black crosses indicate τ_{true}/D combinations that did not result in a peak in the simulated current trace.

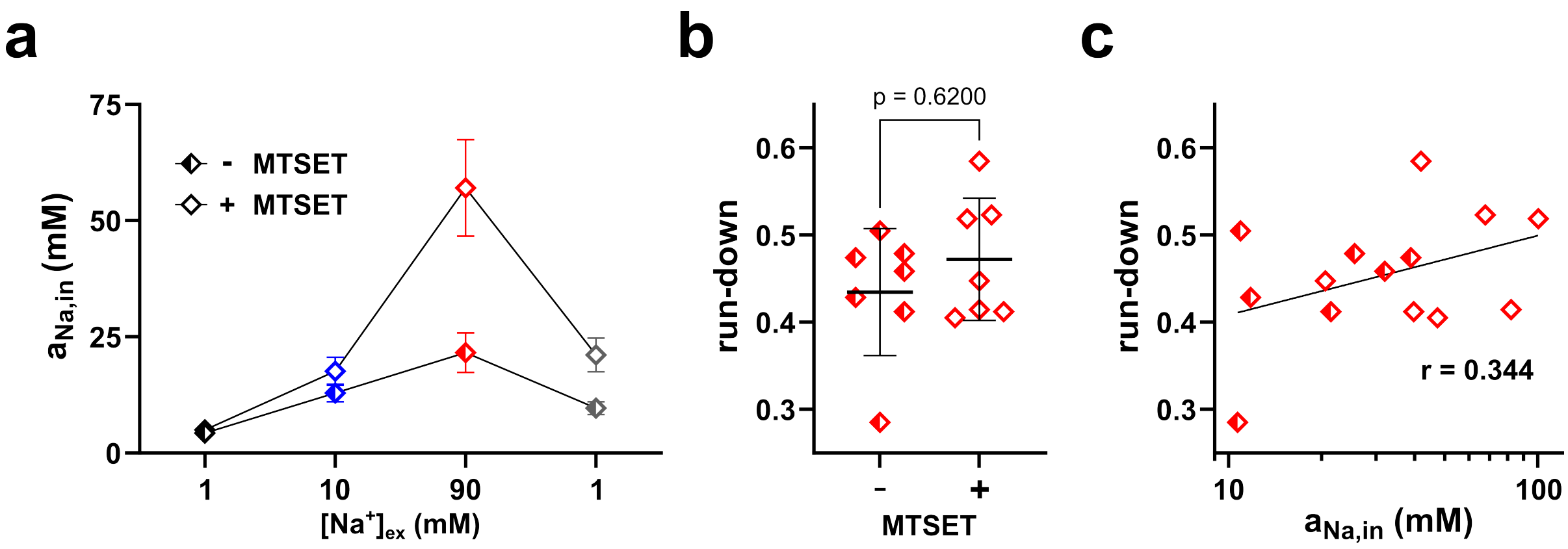

Supplementary Figure S5: Analysis of the relationship between intracellular Na^+ accumulation and current run-down in experiments with oocytes expressing $\alpha\beta_{S520C}\gamma$-ENaC. (a) Mean intracellular Na^+ activities ($a_{Na,in}$) of mock-treated (- MTSET; n = 7, N = 5) and MTSET-treated (+ MTSET; n = 7, N = 4) oocytes expressing $\alpha\beta_{S520C}\gamma$-ENaC from experiments shown in main **Figure 6**. $a_{Na,in}$ values were calculated using **Equation 16** from reversal potentials (E_{rev}) of amiloride-sensitive I/V relationships as shown in **Figure 6c**. **(b)** Current run-down at the end of experiments shown in **Figure 6**. The individual run-down values were calculated from the Na^+ permeabilities (P_{Na}) at $[Na^+]_{ex}$ = 1 mM at the start and the end of each experiment. Statistical comparison was performed using a Mann-Whitney test. **(c)** Scatter plot of $a_{Na,in}$ at $[Na^+]_{ex}$ = 90 mM versus corresponding run-down at the end of the experiments in main **Figure 6**. The solid line represents the best fit of a semi-logarithmic line to the data. The Pearson correlation coefficient (r) provided in the graph indicates a moderate positive correlation between the intracellular Na^+ accumulation and the current run-down observed at the end of each experiment.

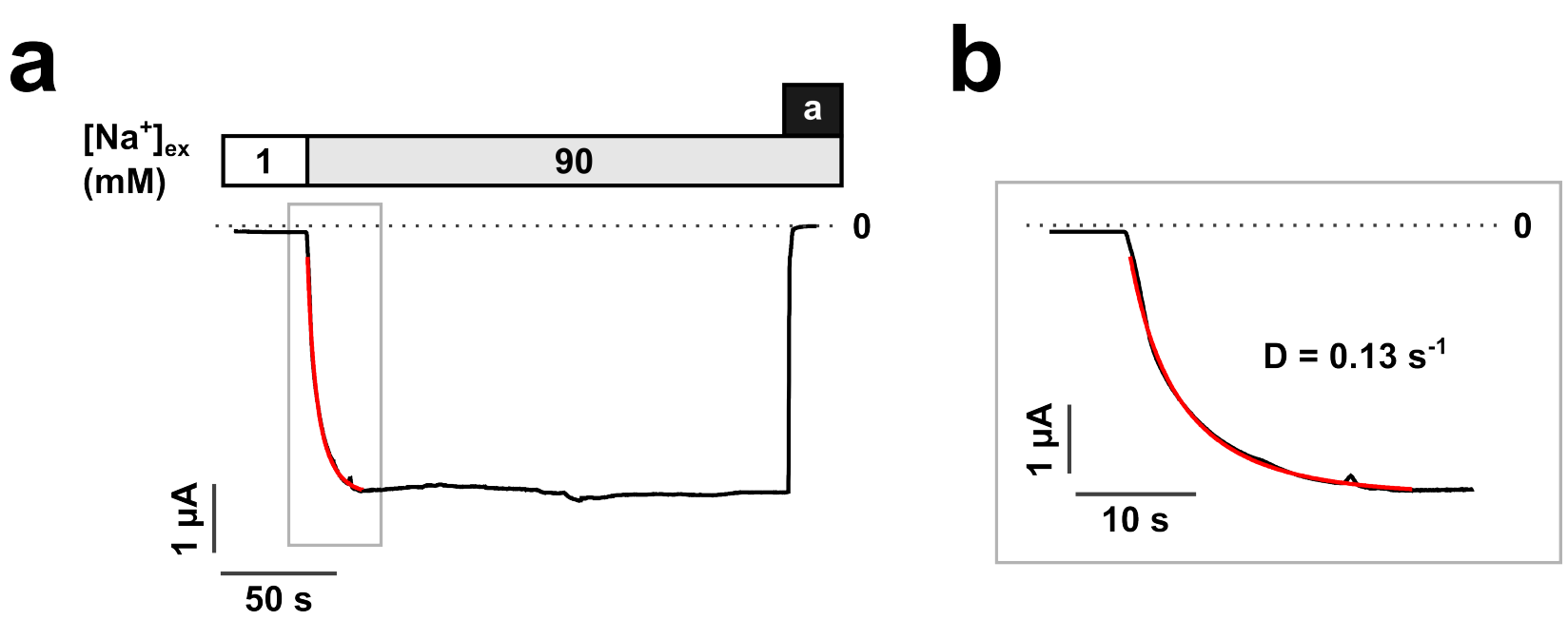

Supplementary Figure S6: Determination of the perfusion rate D by analysing the rising phase of ENaC-mediated transient currents under conditions in which SSI has been abolished. (a) Representative I_M trace from an automated TEVC recording of an oocyte expressing αβγ-ENaC after 5 min treatment with 2 µg/ml chymotrypsin in 1 mM Na^+ solution. The chymotrypsin treatment was carried out to abolish SSI by proteolytic cleavage of ENaC's γ-subunit. Successful elimination of SSI is reflected in the absence of a current peak after the $[Na^+]_{ex}$-jump from 1 mM to 90 mM. $[Na^+]_{ex}$ was changed from 1 mM to 90 mM; 100 µM amiloride (a; black box) was applied at the end of the recording. The solid red line represents the best fit of **Equation A4.3** in combination with **Equation 8** (main text) to the current of the rising phase starting from the inflection point. The rising phase of the current trace is highlighted by a grey rectangle and shown enlarged in **(b)**. The perfusion rate D determined by the shown fit is given in (b).

Appendix S1

Na^+-dependent gating of ENaC can be described with the following two-state Markov-model:

$$\underset{k_{IA}}{\overset{k_{AI}}{A \rightleftharpoons I}}$$

(Scheme A1.1)

In this model, ENaC can exist in an active (Na^+-conductive) state *A* and a Na^+-inhibited (non-conductive) state *I*. The transitions between these two states are determined by the inhibition rate constant k_{AI} and the activation rate constant k_{IA} with k_{AI} depending linearly on $[Na^+]_{ex}$:

$$k_{AI} = k'_{AI} \cdot [Na^+]_{ex}$$

(Equation A1.1)

Due to the $[Na^+]_{ex}$-dependence of k_{AI}, a change in $[Na^+]_{ex}$ will result in a change of the probability to find the channel in the active (P_A) or inhibited (P_I) state. In accordance with Scheme A1.1, the time-dependent change in P_A and P_I can be described by the following first-order differential equations:

$$\frac{dP_A}{dt} = -k'_{AI} \cdot [Na^+]_{ex} \cdot P_A + k_{IA} \cdot P_I$$

(Equation A1.2a)

$$\frac{dP_I}{dt} = k'_{AI} \cdot [Na^+]_{ex} \cdot P_A - k_{IA} \cdot P_I$$

(Equation A1.2b)

Since the channel has to be in state A or state I at any given time t, we can write for the sum of the probabilities P_A and P_I:

$$P_A + P_I = 1$$

(Equation A1.3)

Therefore, we can substitute P_I in **Equation A1.2a** with $1-P_A$:

$$\frac{dP_A}{dt} = -k'_{AI} \cdot [Na^+]_{ex}(t) \cdot P_A(t) + k_{IA} \cdot (1 - P_A(t))$$

(Equation A1.4)

If the change in $[Na^+]_{ex}$ occurs without any time delay, then $[Na^+]_{ex}$ is a constant and **Equation A1.4** can be solved analytically. Integration of Equation A1.4 over the time interval [0,t] yields the following relationship:

$$P_A(t) = \frac{k_{IA}}{(k'_{AI} \cdot [Na^+]_{ex} + k_{IA})} + \left(P_A(0) - \frac{k_{IA}}{(k'_{AI} \cdot [Na^+]_{ex} + k_{IA})}\right) \cdot e^{-(k'_{AI} \cdot [Na^+]_{ex} + k_{IA}) \cdot t}$$

(Equation A1.5)

with *P(0)* being the probability that the channel is in the active state at t = 0.

With the exponential time constant τ_{true}

$$\tau_{true} = \frac{1}{k'_{AI} \cdot [Na^+]_{ex} + k_{IA}}$$

(Equation A1.6)

and the equilibrium probability

$$P_{A,eq} = \lim_{t \to \infty} P_A(t) = \frac{k_{IA}}{k'_{AI} \cdot [Na^+]_{ex} + k_{IA}}$$

(Equation A1.7)

Equation A1.5 can be written as

$$P_A(t) = P_{A,eq} + \left(P_A(0) - P_{A,eq}\right) \cdot e^{-\frac{t}{\tau_{true}}}$$

(Equation A1.8)

Appendix S2

The apparent inhibitory effect of $[Na^+]_{ex}$ on P_{Na} could be caused by the saturation of a Na^+ binding site within the ion permeation pathway. This scenario can be described using the following simple reaction scheme:

$$A^0 + Na^+_{ex} \underset{k_{-1}}{\overset{k_1}{\rightleftharpoons}} A \cdot Na^+ \overset{k_2}{\rightarrow} A^0 + Na^+_{in}$$

where A^0 represents an unoccupied open channel, $A{\cdot}Na^+$ represents an occupied open channel, and Na^+_{ex} and Na^+_{in} are sodium ions in the extra- and intracellular solutions, respectively. Assuming that there is no backflux of Na^+ from the intracellular solution into the channel, the current through the open channel (i) is proportional to the occupancy of occupied channels ($P(A{\cdot}Na^+)$):

$$i = k_2 \cdot P(A \cdot Na^+)$$

(Equation A2.1)

Assuming that the extracellular Na^+ concentration remains unchanged by the ionic current through the channel, $P(A{\cdot}Na^+)$ is given by:

$$P(A \cdot Na^+) = \frac{[Na^+]_{ex}}{K_M + [Na^+]_{ex}}$$

(Equation A2.2)

with the Michaels-Menten constant

$$K_M = \frac{k_{-1} + k_2}{k_1}$$

The Na^+ permeability p_{Na} is defined as

$$p_{Na} = \frac{i}{\Delta[Na^+]}$$

(Equation A2.3)

with $\Delta[Na^+]$ being the Na^+ concentration gradient across them membrane:

$$\Delta[Na^+] = [Na^+]_{ex} - [Na^+]_{in}$$

$[Na^+]_{in} \approx 0$ gives

$$p_{Na} = \frac{i}{[Na^+]_{ex}}$$

(Equation A2.4)

Combination of **Equations A2.1**, **A2.2** and **A2.4** gives

$$p_{Na} = \frac{k_2}{K_M + [Na^+]_{ex}}$$

(Equation A2.5)

For $[Na^+]_{ex} = 0$ **Equation A2.5** yields

$$k_2 = p_{Na}^0 \cdot K_M$$

(Equation A2.6)

Substituting **Equation A2.6** into **A2.5** gives:

$$p_{Na} = p_{Na}^0 \cdot \frac{K_M}{[Na^+]_{ex} + K_M}$$

which is identical to

$$p_{Na} = p_{Na}^0 \cdot \left(1 - \frac{[Na^+]_{ex}}{[Na^+]_{ex} + K_M}\right)$$

(Equation A2.7)

Permeability can therefore be expressed as the product of the maximum permeability p_{Na}^0 at $[Na^+]_{ex} = 0$ and a $[Na^+]_{ex}$-dependent correction factor ε:

$$p_{Na} = p_{Na}^0 \cdot \varepsilon([Na^+]_{ex})$$

(Equation A2.8)

with

$$\varepsilon([Na^+]_{ex}) = 1 - \frac{[Na^+]_{ex}}{[Na^+]_{ex} + K_M}$$

(Equation A2.9)

Equation A2.9 is identical to **Equation 30** in the main text and describes the apparent inhibitory effect of $[Na^+]_{ex}$ on the open channel permeability.

Appendix S3

Steady-state current-voltage relationships recorded from oocytes expressing αβγ-ENaC can be described using the following modified GHK flux equation:

$$I = f_{corr}(t) \cdot P_{Na}^{0} \cdot \left(1 - \frac{[Na^{+}]_{ex}}{[Na^{+}]_{ex} + K_M}\right) \cdot \left(1 - SSI([Na^{+}]_{ex})\right) \cdot \frac{F^2}{R \cdot T} \cdot V_M \cdot \left(\frac{[Na^{+}]_{ex} - [Na^{+}]_{in} \cdot e^{\frac{F}{R \cdot T} \cdot V_M}}{1 - e^{\frac{F}{R \cdot T} \cdot V_M}}\right)$$

(Equation A3.1)

In the absence of SSI (SSI($[Na^+]_{ex}$) = 0) and ENaC current run-down ($f_{corr}(t)$ = 1), this equation simplifies to:

$$I = P_{Na}^{0} \cdot \left(1 - \frac{[Na^{+}]_{ex}}{[Na^{+}]_{ex} + K_M}\right) \cdot \frac{F^2}{R \cdot T} \cdot V_M \cdot \left(\frac{[Na^{+}]_{ex} - [Na^{+}]_{in} \cdot e^{\frac{F}{R \cdot T} \cdot V_M}}{1 - e^{\frac{F}{R \cdot T} \cdot V_M}}\right)$$

(Equation A3.2)

For constant values of $[Na^+]_{in}$ and V_M we can write:

$$I = \left(1 - \frac{[Na^{+}]_{ex}}{[Na^{+}]_{ex} + K_M}\right) \cdot \left(c_1 \cdot [Na^{+}]_{ex} - c_2\right)$$

(Equation A3.3)

with the constants

$$c_1 = \frac{P_{Na}^{0} \cdot F^2 \cdot V_M}{R \cdot T \cdot \left(1 - e^{\frac{F}{R \cdot T} \cdot V_M}\right)}$$

$$c_2 = \frac{P_{Na}^{0} \cdot F^2 \cdot V_M \cdot [Na^{+}]_{in}}{R \cdot T \cdot \left(e^{-\frac{F}{R \cdot T} \cdot V_M} - 1\right)}$$

This equation can be re-written as

$$I = \frac{K_M \cdot \left(c_1 - \frac{c_2}{[Na^{+}]_{ex}}\right)}{1 + \frac{K_M}{[Na^{+}]_{ex}}}$$

(Equation A3.4)

For $[Na^+]_{ex} \rightarrow \infty$, the current amplitude approaches I_{max}:

$$\lim_{[Na^+]_{ex}\to\infty} I = I_{max} = \frac{K_M \cdot (c_1 - 0)}{1 + 0}$$

$$\lim_{[Na^+]_{ex}\to\infty} I = I_{max} = K_M \cdot c_1$$

$$I_{max} = \frac{K_M \cdot P_{Na}^0 \cdot F^2 \cdot V_M}{R \cdot T \cdot \left(1 - e^{\frac{F}{R \cdot T} \cdot V_M}\right)}$$

(Equation A3.5)

Appendix S4

To obtain an estimate of the perfusion rate D in our manual and automated TEVC recordings, we analysed the rising phase of ENaC-mediated transient currents under conditions in which Na^+ self-inhibition has been abolished. This was achieved either by pre-treating oocytes expressing $\alpha\beta_{S520C}\gamma$-ENaC with MTSET (as shown in main **Figure 5b**) or by pre-treating oocytes expressing αβγ-ENaC with 2 µg/ml chymotrypsin (as describes in Material and Methods). Proteolytic cleavage of ENaC‘s γ-subunit by chymotrypsin results in the release of its GRIP (gating relief of inhibition by proteolysis) domain and, consequently, in the elimination of Na^+ self-inhibition (Chraïbi and Horisberger, 2002).

The analysis is based on the following considerations:

The coupling of MTSET to β-Cys520 of $\alpha\beta_{S520C}\gamma$-ENaC (main **Figure 5b,d**) and proteolytic cleavage of αβγ-ENaC (**Supplementary Figure S6**) eliminates the dependence of the open probability on the extracellular Na^+ concentration, allowing us to assume that SSI_{true} equals 0.

Furthermore, we assume that the time interval during which the current rise occurs following the change from $[Na^+]_{ex}$ = 1 mM to $[Na^+]_{ex}$ = 90 mM is sufficiently short that both a change in the intracellular Na^+ concentration and any channel protein turnover can be neglected. Thus, **Equation 19** (main text) simplifies to:

$$I = P_{Na}^0 \cdot \varepsilon([Na^+]_{ex}) \cdot \frac{F^2}{R \cdot T} \cdot V_M \cdot \left(\frac{a_{Na,ex} - a_{Na,in} \cdot e^{\frac{F}{R \cdot T} \cdot V_M}}{1 - e^{\frac{F}{R \cdot T} \cdot V_M}}\right)$$

(Equation A4.1)

We have further assumed that the intracellular Na^+ activity ($a_{Na,in}$) is 4.96 mM, which corresponds to the mean $a_{Na,in}$ value we were able to calculate from the reversal potentials

(E_{rev}) of n = 7 amiloride-sensitive I/V curves of MTSET-treated oocytes expressing $\alpha\beta_{S520C}\gamma$-ENaC at $[Na^+]_{ex}$ = 1 mM (main **Figure 6** & **Supplementary Figure 5a**). $\varepsilon([Na^+]_{ex})$, which accounts for the $[Na^+]_{ex}$-dependence of the open-channel Na^+ permeability, can be calculated using **Equation 30** (main text) with K_M = 73 mM. Using **Equations 20 and 21** (main text), $a_{Na,ex}$ can be calculated as follows:

$$a_{Na,ex} = [Na^+]_{ex} \cdot 10^{-\frac{A\cdot\sqrt{I}}{1+B\cdot r_{Na}\sqrt{I}}}$$

(Equation A4.2)

with A = 0.509 $M^{-1/2}$, B = 3.28 $M^{-1/2}nm^{-1}$ (Christ et al., 1974), r_{Na} = 0.4 nm (Kielland, 1937) and I = 99.5 mM (calculated with **Equation 22** (main text)).

Based on these considerations, the rising phase of the transient current recordings shown in main **Figure 5b** can be described using the following equation:

$$I = P_{Na}^0 \cdot \left(1 - \frac{[Na^+]_{ex}}{[Na^+]_{ex} + K_M}\right) \cdot \frac{F^2}{R\cdot T} \cdot V_M \cdot \left(\frac{[Na^+]_{ex} \cdot 10^{-\frac{A\cdot\sqrt{I}}{1+B\cdot r_{Na}\sqrt{I}}} - a_{Na,in} \cdot e^{\frac{F}{R\cdot T}\cdot V_M}}{1 - e^{\frac{F}{R\cdot T}\cdot V_M}}\right)$$

(Equation A4.3)

Rearranging Equation A4.3 gives:

$$P_{Na}^0 = I \cdot \left[\left(1 - \frac{[Na^+]_{ex}}{[Na^+]_{ex} + K_M}\right) \cdot \frac{F^2}{R\cdot T} \cdot V_M \cdot \left(\frac{[Na^+]_{ex} \cdot 10^{-\frac{A\cdot\sqrt{I}}{1+B\cdot r_{Na}\sqrt{I}}} - a_{Na,in} \cdot e^{\frac{F}{R\cdot T}\cdot V_M}}{1 - e^{\frac{F}{R\cdot T}\cdot V_M}}\right)\right]^{-1}$$

(Equation A4.4)

Equation A4.4 allowed us to calculate P_{Na}^0 for each recording from the current recorded after establishing $[Na^+]_{ex}$ = 90 mM.

The time-dependent change in $[Na^+]_{ex}$ during the rising phase can be described with **Equation 8** (main text):

$$[Na^+]_{ex}(t) = [Na^+]_{ex,eq} - \left([Na^+]_{ex,eq} - [Na^+]_{ex}(0)\right) \cdot e^{-D\cdot t}$$

To calculate D, we fitted **Equation A4.3** in combination with **Equation 8** (main text) to the rising phase of current recordings as shown in main **Figure 5b**. These analyses yielded a perfusion rate of D = 0.33 ± 0.06 s^{-1} (n = 8) in our manual TEVC recordings.

To estimate D of our automated TEVC system, we performed $[Na^+]_{ex}$-jump experiments as shown in main **Figure 1** on $\alpha\beta\gamma$-ENaC expressing oocytes pre-treated with 2 µg/ml

chymotrypsin for 5 min. A representative current trace is shown in **Supplementary Figure S6**. These analyses yielded a perfusion rate of D = 0.19 ± 0.11 s^{-1} (n = 5) in our automated TEVC recordings.

Appendix S5

To estimate the effect of the uncertainty in the correction factor ε on the uncertainty of SSI_{IV}, the standard deviation σ_{SSI} of SSI_{IV} was computed from the variables P^*_{Na} and ε and their respective standard deviations σ_P and σ_ε as follows:

$$\sigma_{SSI} \approx \sqrt{{\sigma_P}^2 \cdot \left(\frac{\partial SSI_{IV}}{\partial {P^*}_{Na}}\right)^2 + {\sigma_\varepsilon}^2 \cdot \left(\frac{\partial SSI_{IV}}{\partial \varepsilon}\right)^2}$$

(Equation A5.1)

with P^*_{Na} being the run-down-corrected relative Na^+-permeability $P'_{Na}([Na^+]_{ex})/P_{Na}(1\ mM)$. Applying **Equation A.5.1** to **Equation 31** (main text) gives:

$$\sigma_{SSI} \approx \sqrt{\left(\frac{\sigma_P}{\varepsilon}\right)^2 + {\sigma_\varepsilon}^2 \cdot \left(\frac{{P^*}_{Na} \cdot \sigma_\varepsilon}{\varepsilon^2}\right)^2}$$

(Equation A5.2)

Here, P^*_{Na} and ε represent the arithmetic means, and σ_P and σ_ε the respective standard deviations.

References

Chraïbi A, Horisberger J-D. 2002. Na self inhibition of human epithelial Na channel: temperature dependence and effect of extracellular proteases. The Journal of general physiology **120**:133–45. DOI: https://doi.org/10.1085/jgp.20028612, PMID: 12149276

Christ C, Hostetler PB, Siebert RM. 1974. Stabilities of calcite and aragonite. Journal of Research of the U.S. Geological Survey **2**.

Kielland J. 1937. Individual Activity Coefficients of Ions in Aqueous Solutions. Journal of the American Chemical Society **59**:1675–1678. DOI: https://doi.org/10.1021/ja01288a032
